# Supplementary material for: Shifting dominant periods in extreme climate impacts under global warming
Source: Nat Commun. 2025 Nov 5;16:9746. doi: 10.1038/s41467-025-65600-7 (PMC12589625; doi:10.1038/s41467-025-65600-7)
Supplement: Supplementary file 1 — Supplementary Information [file 41467_2025_65600_MOESM1_ESM.pdf]

Supplementary Material: Shifting dominant periods in extreme climate impacts under global warming

Karim Zantout[1,2,*], Juraj Balkovic[3], Maik Billing[1], Christian Folberth[3], Simon N. Gosling[4], Tobias Hank[5], Stijn Hantson[6], Toshichika Iizumi[7], Akihiko Ito[8], Jonas Jägermeyr[9,10,1], Atul K. Jain[11], Nikolay Khabarov[12], Sian Kou-Giesbrecht[13], Fang Li[14], Mengxue Li[15,16,17], Tzu-Shun Lin[18], Wenfeng Liu[15,16,17], Christoph Müller[1], Masashi Okada[19], Sebastian Ostberg[1], Kedar Otta[19], Sam Rabin[20,18], Christopher P. O. Reyer[1], Clemens Scheer[20], Julia M. Schneider[5], Florian Zabel[21], Katja Frieler[1], and Jacob Schewe[1]

[1]*Potsdam Institute for Climate Impact Research, Member of the Leibniz Association, Potsdam, Germany*
[2]*Karlsruhe University of Applied Sciences, Karlsruhe, Germany*
[3]*Biodiversity and Natural Resources Program, International Institute for Applied Systems Analysis, Laxenburg, Austria*
[4]*School of Geography, University of Nottingham, Nottingham, UK*
[5]*Department of Geography, Ludwig-Maximilians-Universität, Munich, Germany*
[6]*Facultad de Ciencias Naturales, Universidad del Rosario, Bogotá, Colombia*
[7]*Institute for Agro-Environmental Sciences, National Agriculture and Food Research Organization (NARO), Tsukuba, Japan*
[8]*Graduate School of Agricultural and Life Sciences, The University of Tokyo, 1138657, Tokyo, Japan*
[9]*Center for Climate Systems Research, Columbia Climate School, Columbia University, New York, NY 10025, USA*
[10]*NASA Goddard Institute for Space Studies, New York, NY 10025, USA*
[11]*Department of Climate, Meteorology and Atmospheric Sciences (CLiMAS), University of Illinois, Urbana-Champaign, Urbana, IL 61801, USA*
[12]*Advancing Systems Analysis Program, International Institute for Applied Systems Analysis, Laxenburg, Austria*
[13]*School of Resource and Environmental Management, Simon Fraser University, British Columbia, Burnaby, Canada*
[14]*International Center for Climate and Environment Sciences, Institute of Atmospheric Physics, Chinese Academy of Sciences, Beijing, China*
[15]*State Key Laboratory of Efficient Utilization of Agricultural Water Resources, Beijing 100083, China*
[16]*National Field Scientific Observation and Research Station on Efficient Water Use of Oasis Agriculture in Wuwei of Gansu Province, Wuwei 733000, China*
[17]*Center for Agricultural Water Research in China, College of Water Resources and Civil Engineering, China Agricultural University, Beijing 100083, China*
[18]*NSF National Center for Atmospheric Research, Boulder, CO, USA*
[19]*National Institute for Environmental Studies, Tsukuba, Japan*
[20]*Karlsruhe Institute of Technology (KIT), Institute of Meteorology and Climate Research, Atmospheric Environmental Research (IMK-IFU), Garmisch-Partenkirchen, Germany*
[21]*Department of Environmental Sciences, University of Basel, Basel, Switzerland*
[*]*Corresponding author: Karim Zantout, karim.zantout@pik-potsdam.de*

October 13, 2025

Contents

1 Supplementary Discussion: Robustness tests **2**
1.1 Aggregation level and time window choice 2
1.2 R^2 test 3
1.3 Comparison with observed climate forcing (ISIMIP Phase 3a) 8

2 Supplementary Discussion: Crop resolved dominant period **11**

3 Supplementary Discussion: Correlation analysis **12**

4 Supplementary Discussion: Stochastic test **14**

5 Supplementary Discussion: Alternative wildfire definition **16**
5.1 Wildfire sensitivity analysis 16

6 Supplementary Discussion: Regional event counts **21**

7 Supplementary Discussion: Results for SSP1-2.6 and SSP3-7.0 **25**
7.1 Crop failure 25
7.2 Heatwave 26
7.3 Wildfire 27

8 Supplementary Discussion: Number of contributing models **30**

9 Supplementary Discussion: SSP5-8.5 results for different time windos **35**
9.1 Dominant periods for 2070-2099 35
9.2 Dominant periods for 2050-2099 38

10 Supplementary Discussion: Analysis with $2\Delta T = 250$ years **41**

11 Supplementary Discussion: Comparison to autoregressive model **43**

12 Supplementary Discussion: Standard deviation of dominant periods **44**

1 Supplementary Discussion: Robustness tests

1.1 Aggregation level and time window choice

In order to investigate the stability of our results we calculate the median dominant return period aggregated over all t_0 and models for the picontrol simulation with $2\Delta T = 50$ years (see Fig. 1 in the main text) and compare to the same result where the aggregation is only over all models at $t_0 = 1850$ (Supplementary Fig. 1).

The totally aggregated results are consistent with results for $t_0 = 1850$ with minor differences due to the snapshot character at $t_0 = 1850$ compared to the full time aggregation. For example, we find a similar distribution of dominant periods with some clusters of 4-7 years dominant periods in Australia in the case of crop failure that are replaced with the regionally more consistent 10-13 years dominant period in the totally aggregated result. Similarly, we observe heatwave clusters with dominant periods 7-13 years across all world regions (see Supplementary Fig. 1 b) that do not persist in the aggregated picture. For wildfires, we find in general more dominant periods at $t_0 = 1850$ (see Supplementary Fig. 1 c) which do not appear consistent along all time windows and therefore disappear in the time aggregated result (see Fig. 1 in the main text). These calculations indicate that the totally aggregated results in the main text average out minor difference that appear in a snapshot of dominant periods at $t_0 = 1850$.

Additionally, we show the same results where the aggregation is over all

$$t_0 = 1850,\ 1900,\ 1950,\ 2000,\ 2040$$

and models for $2\Delta T = 60$ years. If we consider the original approach we find dominant periods below 1% in all impact categories for $2\Delta T = 60$y due to minor trends that accumulate for the larger time series. To compensate for the time trend we remove the linear trend from the correlation function as described in Sec. 2 in the main text. Note that linear detrending only introduces minor changes in the case of $2\Delta T = 50$y (see Supplementary Fig. 2). For the detrended result with $2\Delta T = 60$ years we find mostly the same dominant return periods compared to $2\Delta T = 50$ years but significantly fewer dominant periods (see Supplementary Fig. 3). Note that there is a minor shift in dominant periods due to a shift of Fourier frequencies when moving from $2\Delta T = 50$ years to 60 years. Specifically, the detectable dominant periods are $\Delta T/n$ with $n = 1, \ldots, \Delta T$ that can reassemble into more frequencies within the multi-model aggregation. The main difference is the suppressed regularity in the case of crop failure. Defining crop failures through the 97.5th percentile of an underlying distribution (see Sec. 4 in the main text) allows for only few occurrences in the time series data which leads to a weaker signal if the time range is extended because regularity has to be preserved over a longer time range.

In conclusion, the analysis shows that differences of the dominant period results are stabilized through aggregation over all time windows. The results are only sensitive in terms of the regularity to irregularity ratio where irregularity can increase when changing the time window as either spectral leakage suppresses clear regularity signals or extending the time window inhibits regularity through temporal decorrelation. As a consequence we suggest - as for all related time series analysis tools - to be cautious when choosing the parameters of the Fourier decomposition to ensure both a strong signal and stable dominant periods.

1.2 R^2 test

Within our methodology we fixed a threshold for the quality of the Fourier fit in the definition of the dominant period in terms of an adjusted R^2 threshold of $R^2 > 0.5$ (see Sec. 4 in the main text). The reasoning is that a dominant period has to explain the majority of the time series variation. We show in how far our results change as a function of adjusted R^2 through the median dominant periods for crop failure over all t_0 and models under pre-industrial climate conditions in Supplementary Fig. 4.

We mainly observe no significant qualitative changes in the regional distribution and a reduction/enhancement of the regularity signal when increasing/reducing the adjusted R^2 to 0.6 and 0.4, respectively. This effect is again due to the strict crop failure definition that makes it difficult to observe regularity patterns when the quality requirement of the Fourier fit is increased. Furthermore, the reduction of the R^2 threshold leads to a relative and absolute increase of dominant periods between 7-10 years but no significant new dominant period signals. We have checked that heatwave and wildfire results are even less strongly affected by varying R^2.

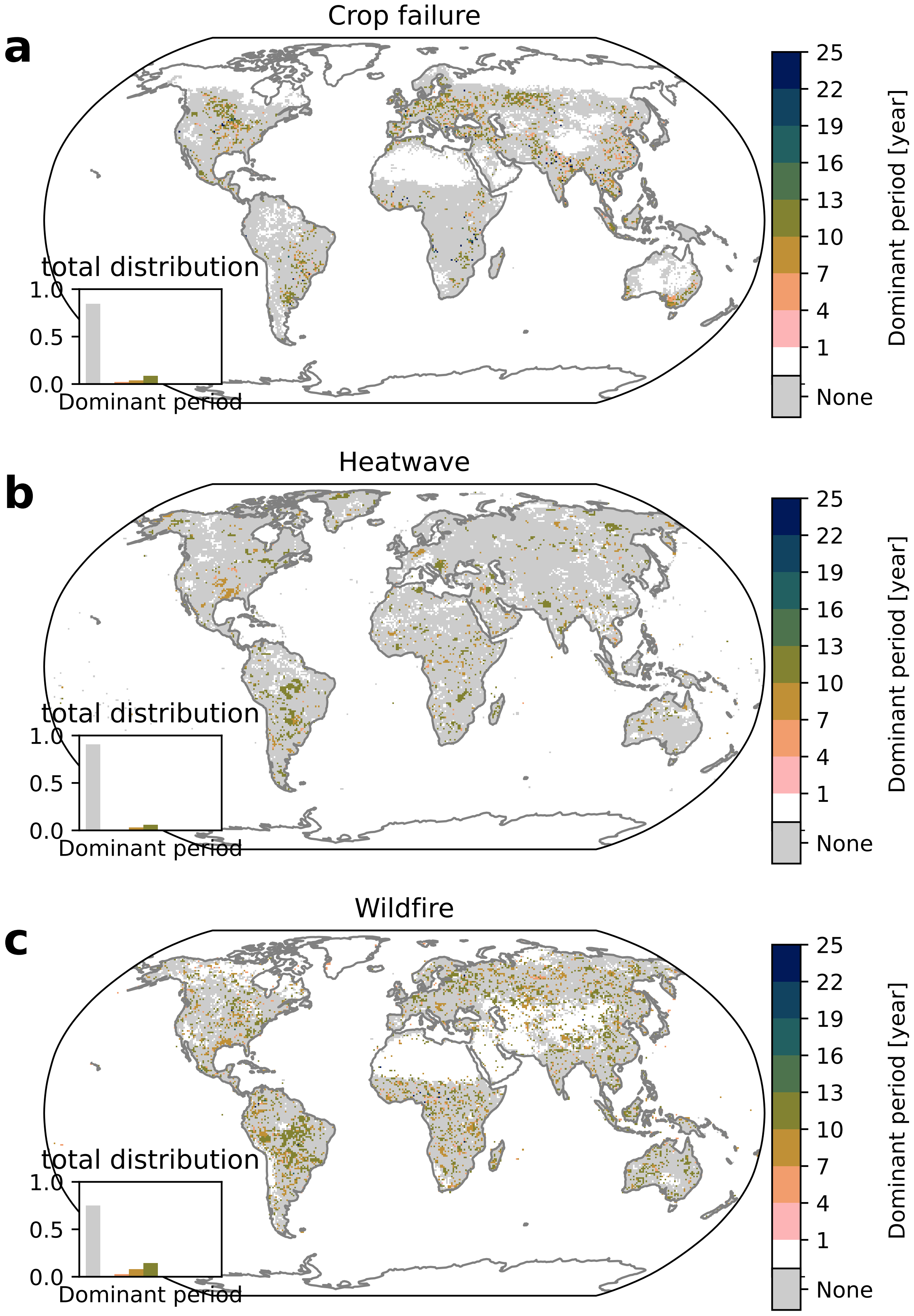

Supplementary Figure 1: **Dominant periods at $t_0 = 1850$.** Median dominant return period for $2\Delta T = 50$ years over all models at $t_0 = 1850$ for (**a**) crop failure, (**b**) heatwave and (**c**) wildfire. The white color signifies no extreme climate impact occurrence and gray color signifies no dominant period (irregularity) while existing dominant periods are grouped in three-year regularity intervals ranging from 1-4 years (pink) to 22-25 years (blue). The inset shows the distribution of the dominant period counts.

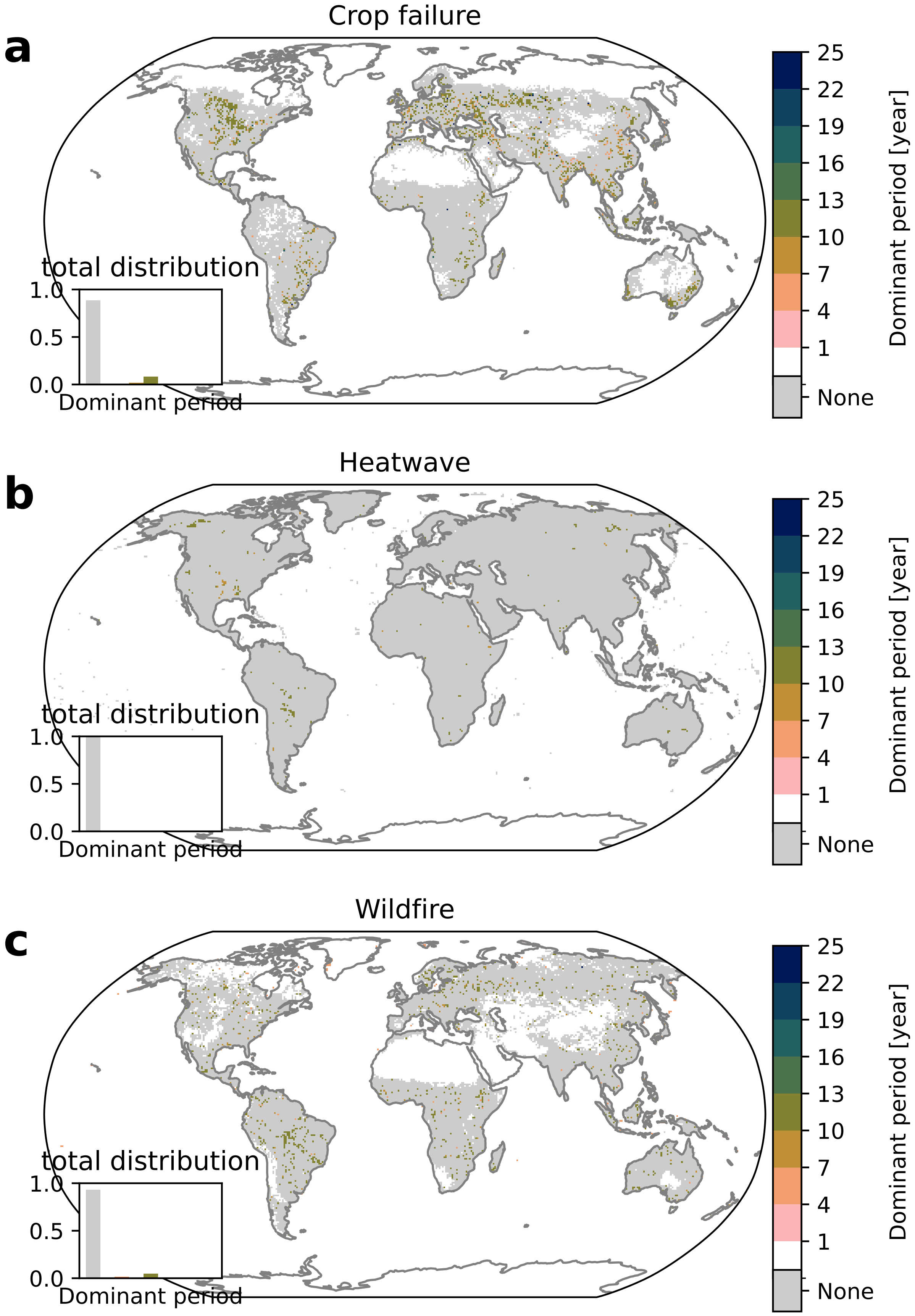

Supplementary Figure 2: **Dominant periods for $2\Delta T = 50$y (linearly detrended).** Median dominant return period from linearly detrended correlation function for $2\Delta T = 50$ years over all models and $t_0 = 1850,\ 1900,\ 1950,\ 2000,\ 2050$ for (**a**) crop failure, (**b**) heatwave and (**c**) wildfire. The white color signifies no extreme climate impact occurrence and gray color signifies no dominant period (irregularity) while existing dominant periods are grouped in three-year regularity intervals ranging from 1-4 years (pink) to 22-25 years (blue). The inset shows the distribution of the dominant period counts.

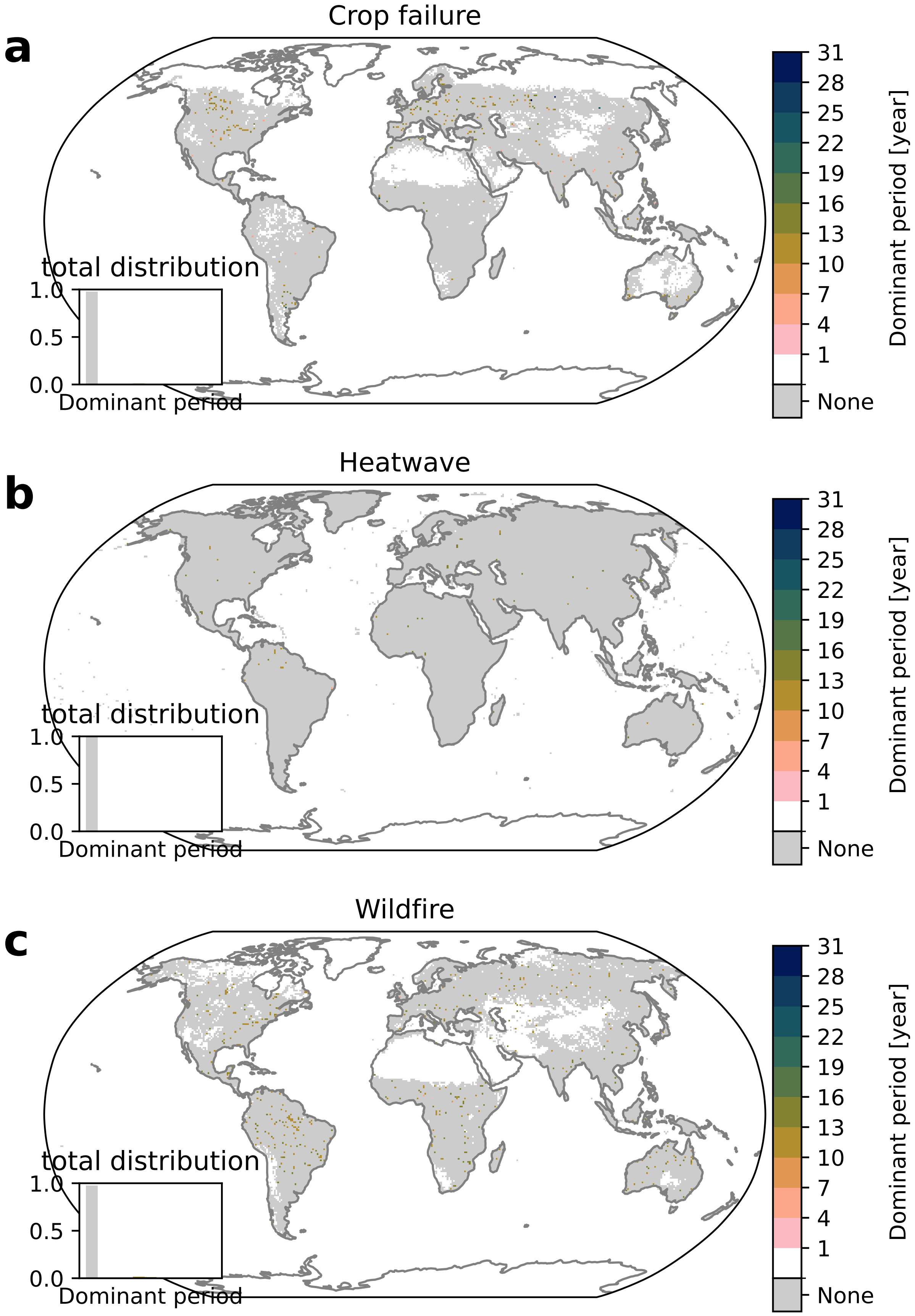

Supplementary Figure 3: **Dominant periods for $2\Delta T = 60$y (linearly detrended).** Median dominant return period for $2\Delta T = 60$ years over all models and $t_0 = 1850,\ 1900,\ 1950,\ 2000,\ 2040$ for (**a**) crop failure, (**b**) heatwave and (**c**) wildfire. The white color signifies no extreme climate impact occurrence and gray color signifies no dominant period (irregularity) while existing dominant periods are grouped in three-year regularity intervals ranging from 1-4 years (pink) to 22-25 years (blue). The inset shows the distribution of the dominant period counts.

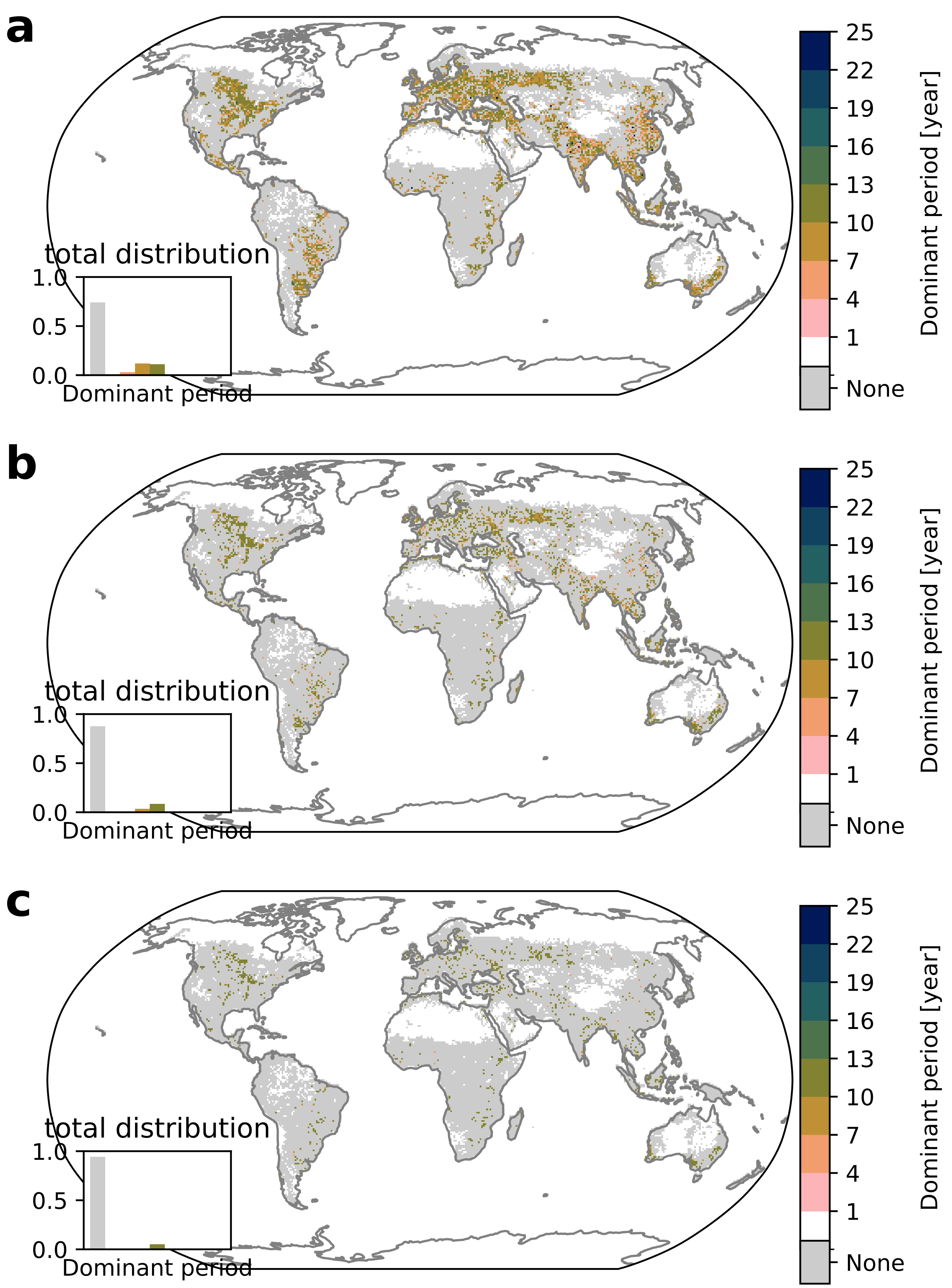

Supplementary Figure 4: **Median dominant period for** $R^2 = 0.4,\ 0.5,\ 0.6$**.** Median dominant period for crop failure and $2\Delta T = 50$ years over all models and t_0 for (**a**) adjusted $R^2 = 0.4$, (**b**) 0.5, and (**c**) 0.6.

	observations	impact model
crop failure	GSWP3-W5E5	ACEA, CROVER, CYGMA1p74, EPIC-IIASA, ISAM, LDNDC, LPJmL, pDSSAT, PEPIC PROMET
heatwave	20CRV3, 20CRV3-ERA5, 20CRV3-W5E5, GSWP3-W5E5	HWMID
wildfire	GSWP3-W5E5	CLASSIC, LPJmL5-7-10-FIRE, VISIT

Supplementary Table 1: Table of climate and impact models used for ISIMIP Phase 3a calculations.

1.3 Comparison with observed climate forcing (ISIMIP Phase 3a)

In order to provide further evidence for the climatic oscillations as source of the regularities and exclude artifacts from GCMs as sources for our results e.g., drifts [1] or deviations in global climate oscillation modes [2], we present here results based on ISIMIP Phase 3a [3]. The fundamental difference to the analysis in the main article consists of observational climate related forcings instead of GCMs. In Supplementary Tab. 1 we name the observations and impact models that provide inputs to the subsequent results.

While in the case of crop failure we have the same impact models and additionally ACEA and pDSSAT, we have the same impact models for heatwaves and wildfire. Moreover, we can base our heatwave analysis on four different global meteorological forcing data (see details in Ref. [3]). In this observation based study we have to limit the parameters of our time series analysis tool, namely

$$t_0 \in \{1901, 1950\}, \;\; 2\Delta T = 50 \text{ years.} \tag{1}$$

As the resulting time windows contain climate forcing trends we use detrended climate forcing [3] except for the case of crop failure where no detrended results are available. The resulting median dominant periods over all t_0 and models are shown in Supplementary Fig. 5 for the ISIMIP3a results and in Supplementary Fig. 6 for ISIMIP3b results.

The main difference between the picontrol results from GCMs and the dominant periods from observation based climate forcing is the higher irregularity in the case of crop failure and wildfire in ISIMIP3a. The main reasons for the increased irregularity is the reduced number of climate observations compared to climate models which allows to compensate effects of spectral leakage (see Sec. 4). This exercise shows not only that our results can be found in observation based climate forcing but also that the distribution of dominant periods is sufficiently well represented in GCMs to further investigate future trends.

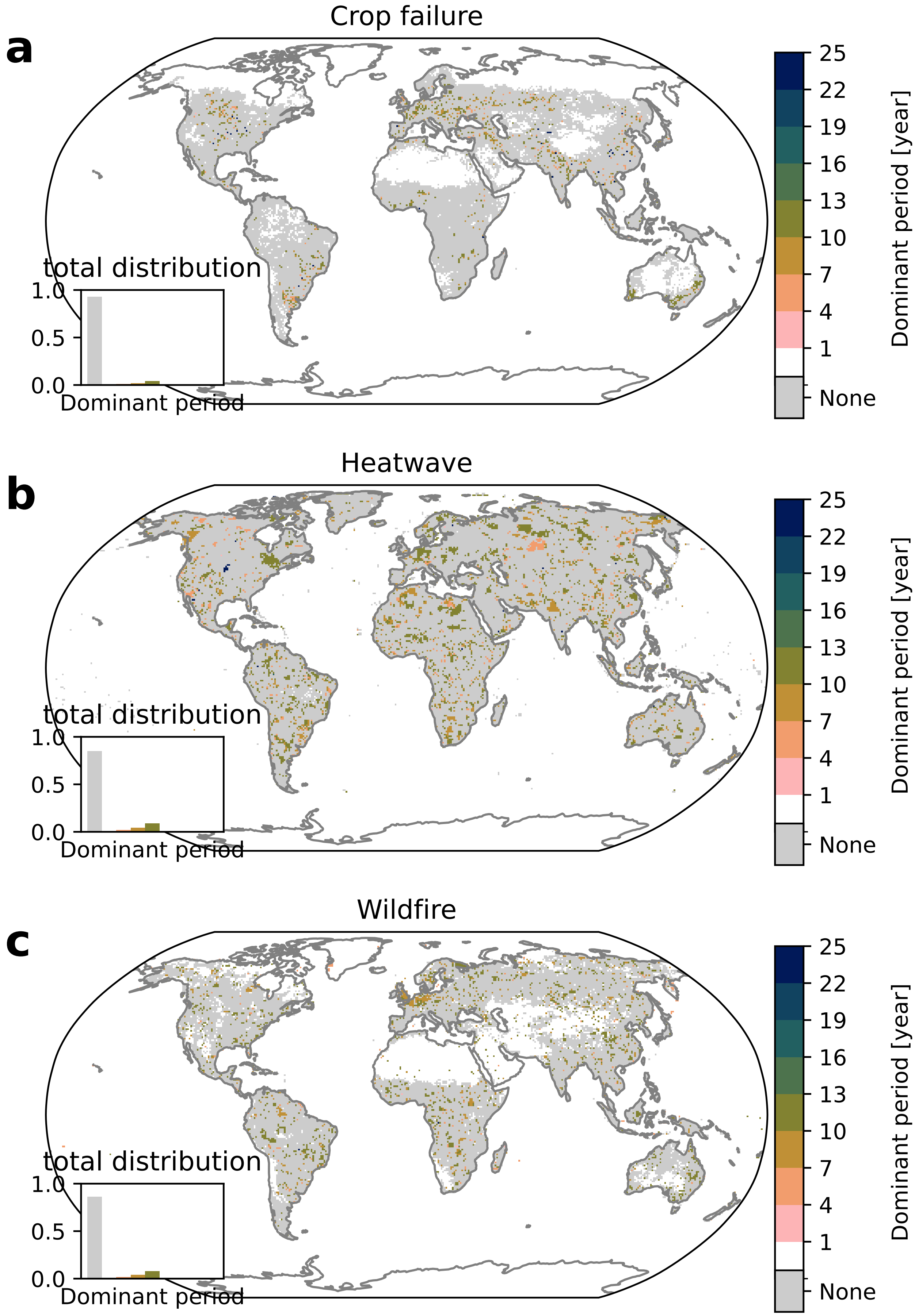

Supplementary Figure 5: **Median dominant period for ISIMIP3a.** Median dominant return period for $2\Delta T = 50$ years and $t_0 \in \{1901, 1950\}$ over all models and t_0 for (**a**) crop failure, (**b**) heatwave, and (**c**) wildfire for ISIMIP3a.

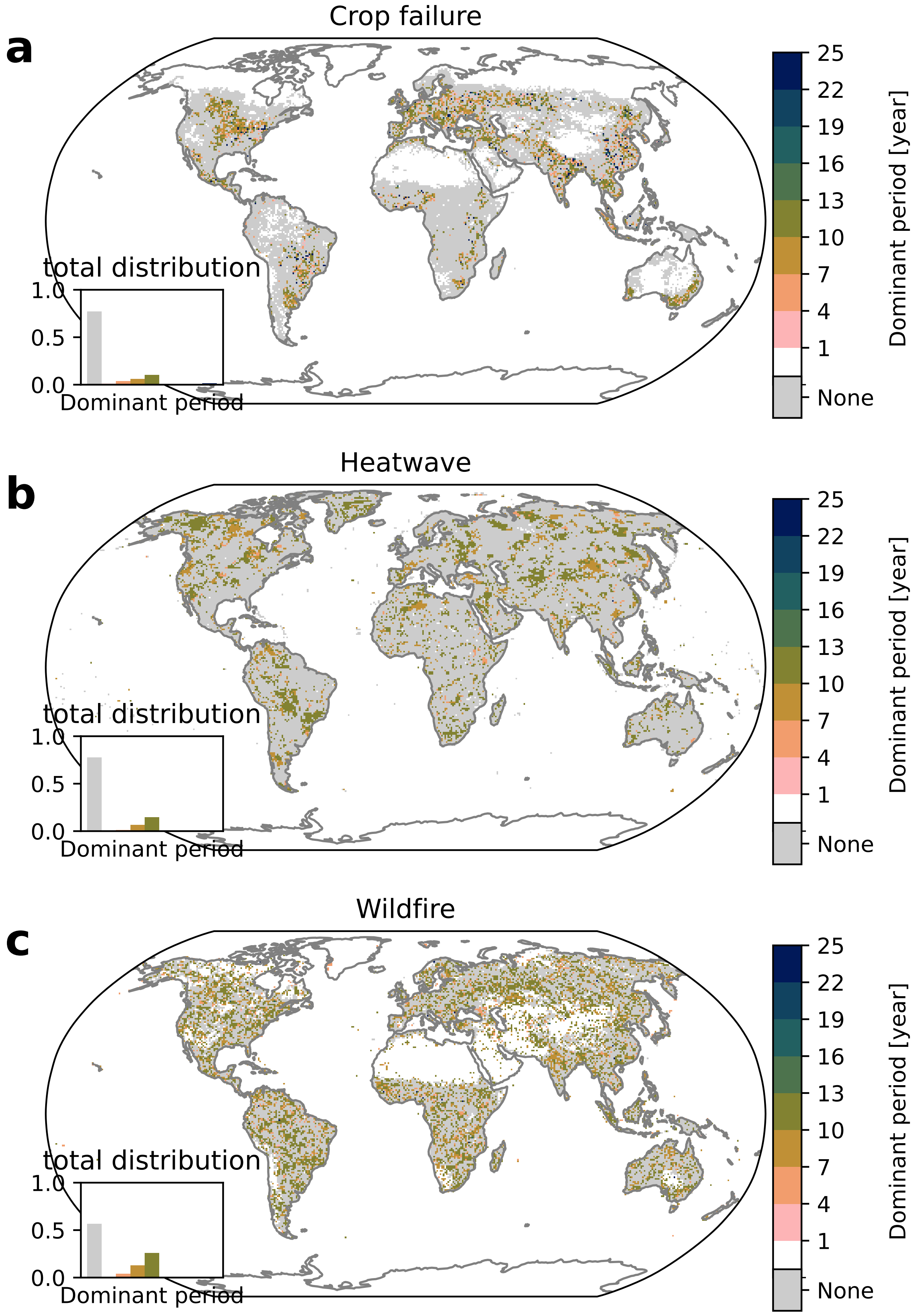

Supplementary Figure 6: **Median dominant period (ISIMIP3b) for ISIMIP3a comparison.** Median dominant return period for $2\Delta T = 50$ years and $t_0 \in \{1901, 1950\}$ over all models and t_0 for (**a**) crop failure, (**b**) heatwave, and (**c**) wildfire for ISIMIP3b.

2 Supplementary Discussion: Crop resolved dominant period

Since crop yields are differently affected by climate modes [4–6] we calculate dominant periods for each crop type separately.

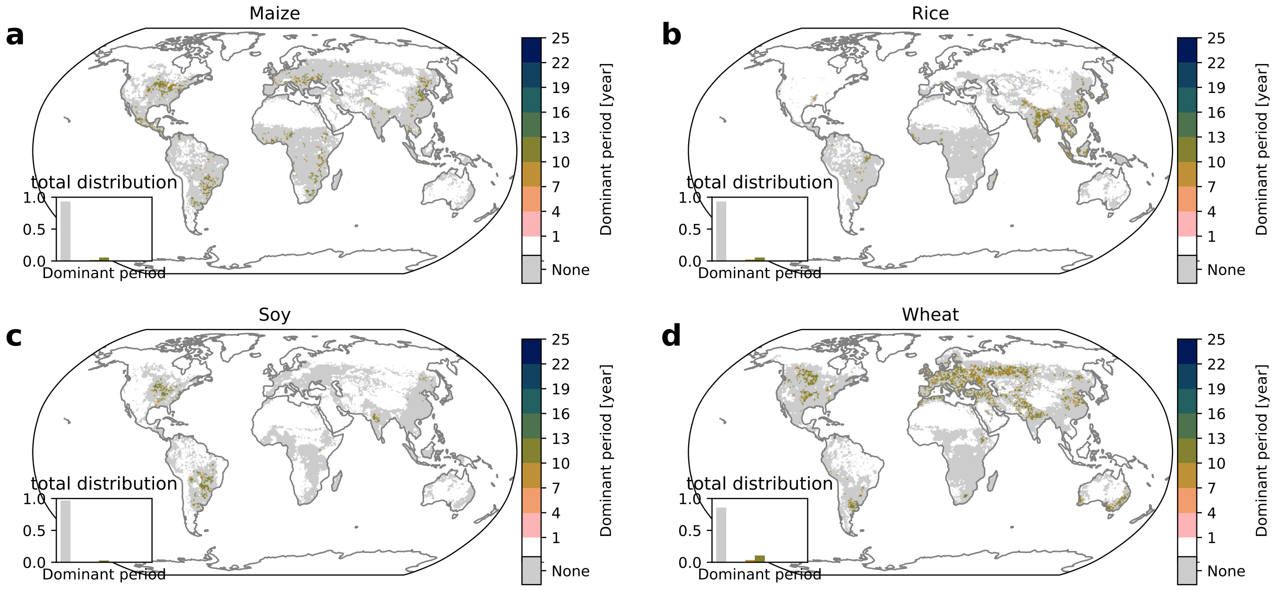

Supplementary Figure 7: **Crop resolved dominant periods.** Median dominant period for (**a**) maize, (**b**) rice, (**c**) soy, and (**d**) wheat crop failure for picontrol aggregated over all time windows 1850-1899, 1900-1949, ..., 2050-2099 and climate-impact models. The white color signifies no extreme climate impact occurrence and gray color signifies no dominant period (irregularity) while existing dominant periods are grouped in three-year regularity intervals ranging from 1-4 years (pink) to 22-25 years (blue). The inset shows the distribution of the dominant period counts.

We find the same dominant periods across all crop types (see Supplementary Fig. 7) and consistent with the totally aggregated result in the main text Fig. 1 a. The main difference between crop type is the regional distribution of dominant periods. For example, dominant periods for maize failure occur on all continents while for rice we have a concentration in South America and South/South East Asia in agreement with [6]. In the case of soy we find dominant periods mainly in the Americas whereas yield fluctuation influences from ENSO are reported in more world regions [6]. Similarly, we find dominant periods for wheat failure across all world regions while reported influences from ENSO are fewer in Europe and Asia [6].

3 Supplementary Discussion: Correlation analysis

To investigate the connection between ENSO and the different extreme event categories we perform a correlation analysis. We use the annual extreme event affected area from historical ISIMIP3a simulations (see Supplementary Material Sec. 1.3) and the yearly-mean Darwin Southern Oscillation Index (SOI) based on annual standardization [7]. Note that this choice is due to quality issues from Tahiti data before 1935 [7].

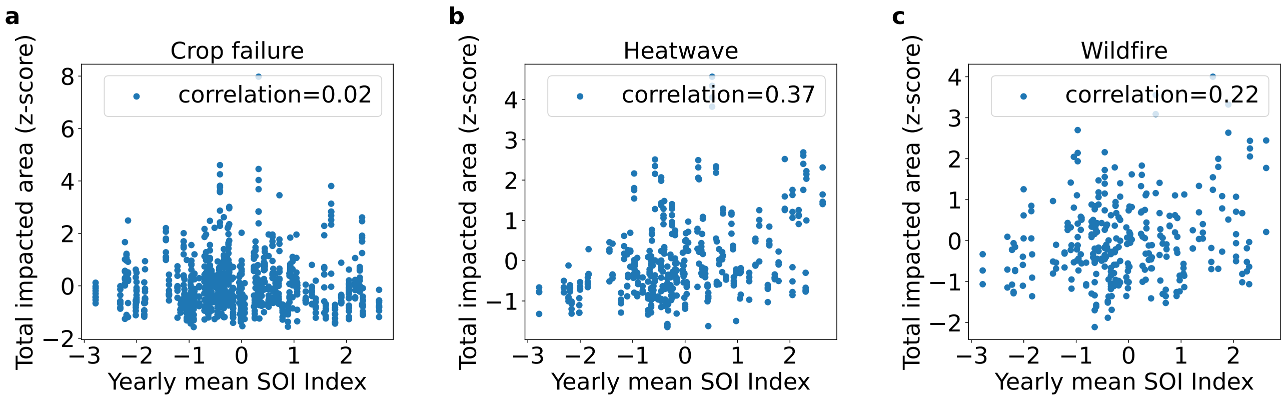

Supplementary Figure 8: **Correlation between extreme event and Southern Oscillation Index.** Pearson correlation between historical annual extreme event affected area from ISIMIP3a simulations and yearly-mean Darwin Southern Oscillation Index (SOI) for (**a**) crop failure, (**b**) heatwave, and (**c**) wildfire.

As expected heatwaves show the strongest correlation (0.37) with ENSO while for wildfires we observe a slightly smaller value of 0.22 (see Supplementary Fig. 8 b and c). On the other hand, for crop failure we find no correlation (0.02) which is due to the strong region and crop type dependence on climate variations [4, 5] (see Supplementary Fig. 8 a).

By calculating crop type specific correlations, we obtain a clearer signal with positive model-median correlation for maize and negative model-median correlation for wheat in agreement with studies on crop yield variability [8] (see Supplementary Fig. 9). In the case of rice and soy we find a model-median positive and near-zero correlation, respectively, which is opposite to the crop yield variability study from Ref. [8] where crop yields are considered.

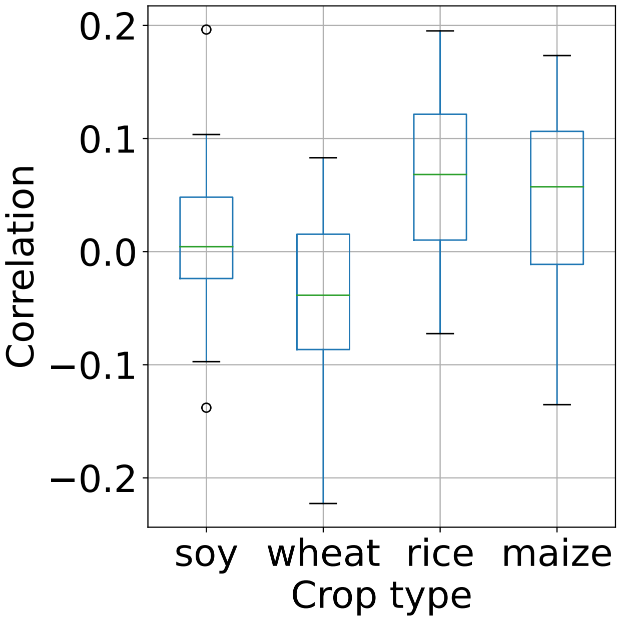

Supplementary Figure 9: **Crop resolved correlation.** Crop resolved Pearson correlation between historical annual crop failure affected area from ISIMIP3a simulations and yearly-mean Darwin Southern Oscillation Index (SOI). The horizontal line signifies the median value while the box boundaries signify the first (Q1) and third (Q3) quartile while the whiskers signify the range Q1$\pm$1.5 IQR, where IQR is the inter-quartile range.

4 Supplementary Discussion: Stochastic test

The observed dominant return periods in the pre-industrial climate scenario may be a result of natural processes but to exclude a stochastic explanation we perform a statistical test. Based on the time-series data $f_i^{jk}(\cdot,\theta,\phi)$, where f is the affected area by climate impact i under climate and impact model j, k, respectively, and θ,ϕ are the grid cell location, we calculate the respective impact probability through the relative frequency, namely

$$p_i^{jk}(\theta,\phi) = \frac{\sum_{t=1850}^{2100} \mathbb{I}_{(0,1]}\left(f_i^{jk}(t,\theta,\phi)\right)}{251}, \tag{2}$$

where $\mathbb{I}$ is the indicator function. We sample from these impact probabilities a random time series for $t \in \{1850, 1851, \dots, 2100\}$. In the case of wild fires we model

$$\hat{f}^{jk}_{\text{wildfire}}(\cdot,\theta,\phi) \sim \text{Bern}(p^{jk}_{\text{wildfire}}(\theta,\phi)), \tag{3}$$

where $\hat{f}$ is the randomly generated time series, Bern(p) is the Bernoulli distribution with success probability p. For crop failure we compute the failure probability for each crop and irrigation type separately and multiply by the land use fraction for each grid cell before summing the individual crop failure times series. In mathematical terms, we define

$$\hat{f}^{jk}_{\text{crop failure}}(\cdot,\theta,\phi) = \sum_i X^{jk}_{i,\text{crop failure}}(\cdot,\theta,\phi) \cdot l_i(\theta,\phi), \tag{4}$$

$$X^{jk}_{i,\text{crop failure}}(\cdot,\theta,\phi) \sim \text{Bern}(p^{jk}_{i,\text{crop failure}}(\theta,\phi)), \tag{5}$$

where i runs over all crop and irrigation types and $l_i(\theta,\phi)$ is the land use fraction for crop and irrigation type i at grid cell position (θ,ϕ). Finally, in the case of heatwaves we simply sample from the Bernoulli distribution,

$$\hat{f}^{jk}_{\text{heatwave}}(\cdot,\theta,\phi) \sim \text{Bern}(p^{jk}_{\text{heatwave}}(\theta,\phi)). \tag{6}$$

Through this experimental setup we exclude contributions to regularity in the time series that stem from the definition of the extreme event (e.g. land use shares). From these randomly generated time series we derive the dominant return periods for all t_0 and all climate and impact models at each grid cell. For comparison with actual data we compute the median value, mean value and standard deviation over all t_0,θ,ϕ,j and k and compare these values to the randomly sampled results that were converged with 10000 samples (see Supplementary Fig. 10). In addition, we also show the signal ratio that is defined as ratio between number of time series with dominant return period and number of non-zero time series in the interval $[t_0, t_0 + DT)$.

We find that for all impact types there is at least one statistical value that lies outside the 2.5th to 97.5th percentile range of the simulated results. In the case of wild fires the differences are visible in all statistics (see Supplementary Fig 10). These large deviations are due to the fire dynamics that are present in the impact models and cannot be reproduced through probabilistic sampling. For crop failure we find the large deviations for the median, mean, and standard deviation due to the crop yield dynamics is not being represented in random samplings. Only for heatwaves we find that the statistical test yields close results but the standard deviation and mean value are below the 2.5th percentile and above the 97.5th percentile, respectively. The reduced discrepancy for heatwaves is related to the direct effect from GCMs which makes it difficult to observe non-trivial regular time series (see Sec. 2 discussion in main text). In summary, we conclude that the observed time regularities are significantly different from a purely random data setup and related to underlying the climate-impact model.

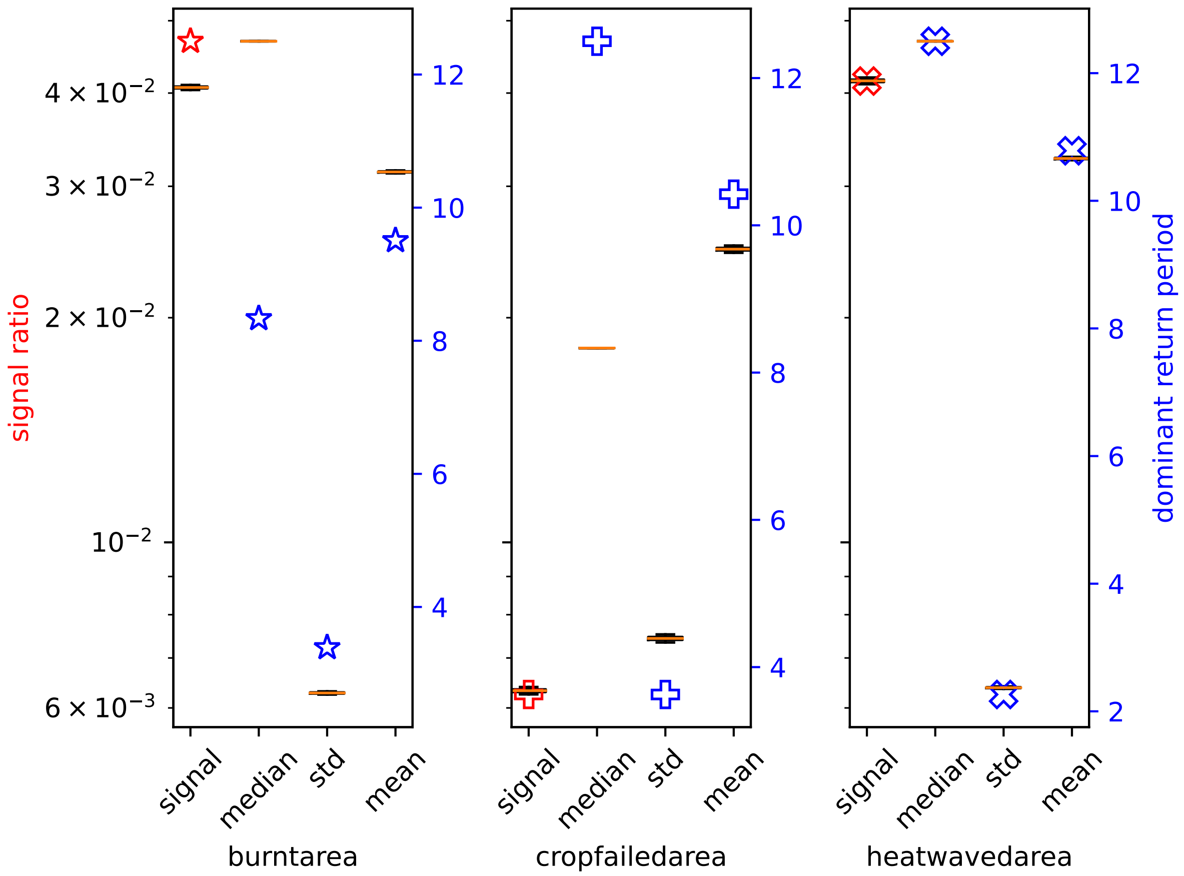

Supplementary Figure 10: **Results of dominant period statistical test.** Results of the statistical test for each impact type. The box plots show the results from the stochastic model while the stars, crosses, and pluses represent the actual results. The whiskers denote the 2.5th and 97.5th percentiles.

5 Supplementary Discussion: Alternative wildfire definition

In contrast to the wildfire definition applied in the main text we present here a different definition to extract the subdominant vegetation growth dynamics which is suppressed when considering only extreme wildfires. We transform the burnt area time series by introducing a cap to restrain wildfires to similar order of magnitudes. Moreover, this also leads to an effective limitation of the wildfire dynamics to the temporal variation below that cap. The fire dynamics is therefore not determined by all wildfire sizes; this would lead to an overlap of several dominant periods. Instead the transformed time series only contains temporal variations from wildfires on smaller scales. The value of the annual burnt area cap itself is determined by studying the change in dominant period patterns as a function of the cap value (see Sec. 5.1).

We extract the temporal dynamics of wildfire emergence by defining wildfire exposure as annual burnt area and capping the burnt area at small values, e.g. 1-2% of the affected grid cell area (see Supplementary Fig. 13). Note that we do not apply the red noise (AR(1) model) test in this wildfire classification (see Sec. 4 in the main text) since an AR(1) noise model is not sufficient to account for the long time scales of regrowth dynamics. For a cap of 1%, we observe more regularity in wildfires than irregularity with 10% of all impacted grid cells exhibiting no regularity (see Supplementary Fig. 13 a). Wildfires appear mostly in two groups of dominant periods, namely with long dominant periods of 22 years or even higher (80% of affected grid cells) and high variability with a dominant period of 1-4 years (6% of affected grid cells). Note that for time series of 50 years length, a strong trend on long timescales cannot be distinguished from a periodic signal with dominant period 25 years.

While the high-frequency regions are in Sub-Saharan Africa, the Iberian peninsula, in the east of South America, the north of Australia, and the Middle East, we find low-frequency regions in other world regions, e.g., in South America except the northern Cerrado and major parts of Australia. Note that high dominant frequencies are not necessarily extreme but normal in some world regions, e.g. in savanna regions where large fires can occur annually [9]. Our results are consistent with the global distribution of observed fire regimes [10] with the exception around the Gulf of Mexico and central and southeast Asia, where observation data suggests high frequency wildfires while our results show large dominant periods. Note that this does not mean that the subdominant regularity signals cannot be high frequency. The observed fire regimes as well as our modeled results are determined by the regrowth rates of the local biomes [10–12]. In grassland and savannahs, vegetation regrows quickly, so that fuel is restored within a short time after a fire, and frequent fires of similar intensity are a natural feature of such biomes. In contrast, boreal forests take much longer to regrow, such that after a fire fuel availability for a new fire of the same intensity remains limited for many years or even decades. Note that the relationship between wildfires and climate modes such as ENSO [13] is not visible here since the full time series variation is capped at 1% to extract the emergence dynamics of wildfires. Our wildfire classification in the main text takes the full time series variation into account and indeed reveals climate mode signals.

5.1 Wildfire sensitivity analysis

In this section we investigate the sensitivity of the median wildfire dominant periods towards the choice of the wildfire cap. In the previous section we analyzed cap values of 1-2% to extract the dynamics of wildfire emergence. We first consider the extreme case where the cap is 0.5% (see Supplementary Fig. 11 a) observing largely extended highest frequency regions compared to the 1% result (see Supplementary Fig. 13 a) signifying a trend towards the limiting case where any non-zero wildfire time series leads to a dominant period of 1 year.

In the other two cases where the cap is 50% and 100% (see Supplementary Fig. 11 b and c) we mostly find irregularity and largest dominant periods. This is due to the mixing of several time scales

in wildfire data where only largest time scale signals survive while all smaller time scales are made incoherent.

Moving away from these two boundary cases we find emerging highest frequency regimes when reducing the cap from 20% to 10% and 5% (see Supplementary Fig. 12).

This emergence of regularity occurs at the cost of previously irregular signals since capping the wildfire affected area essentially restricts large time scales and therefore generates increased coherence. Finally, we find the applied cap yields stable results when increasing the cap size by 50% and even 100%, namely cap sizes of 1.5% and 2% (see Supplementary Fig. 13).

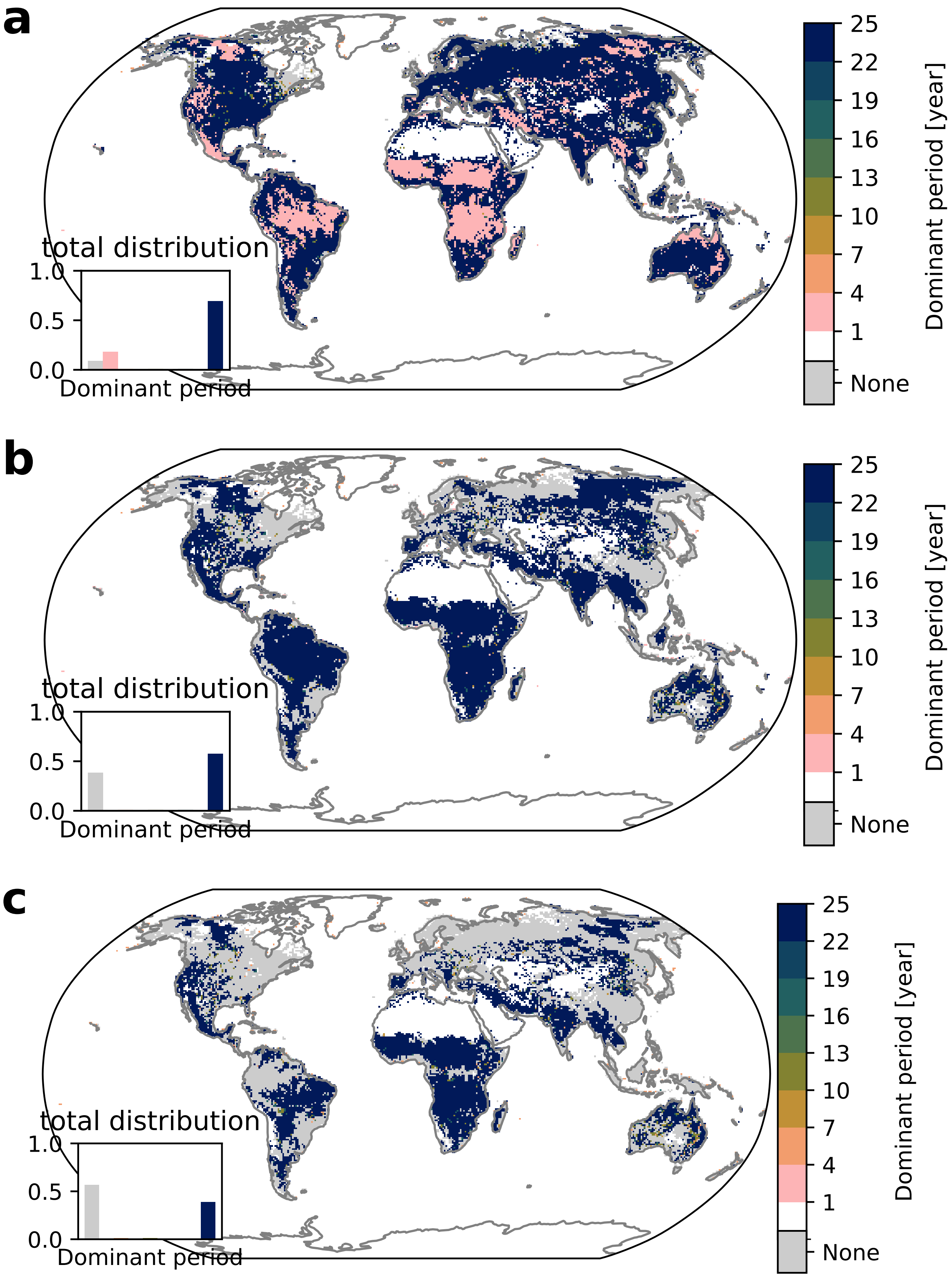

Supplementary Figure 11: **Dominant periods for wildfire caps I.** Median dominant period of wildfires for a wildfire cap of (**a**) 0.5%, (**b**) 50%, and (**c**) 100%. The gray color signifies no dominant period (irregularity) while existing dominant periods are grouped in three-year intervals ranging from 1-4 years (pink) to 22-25 years (blue). The inset shows the distribution of the dominant period counts.

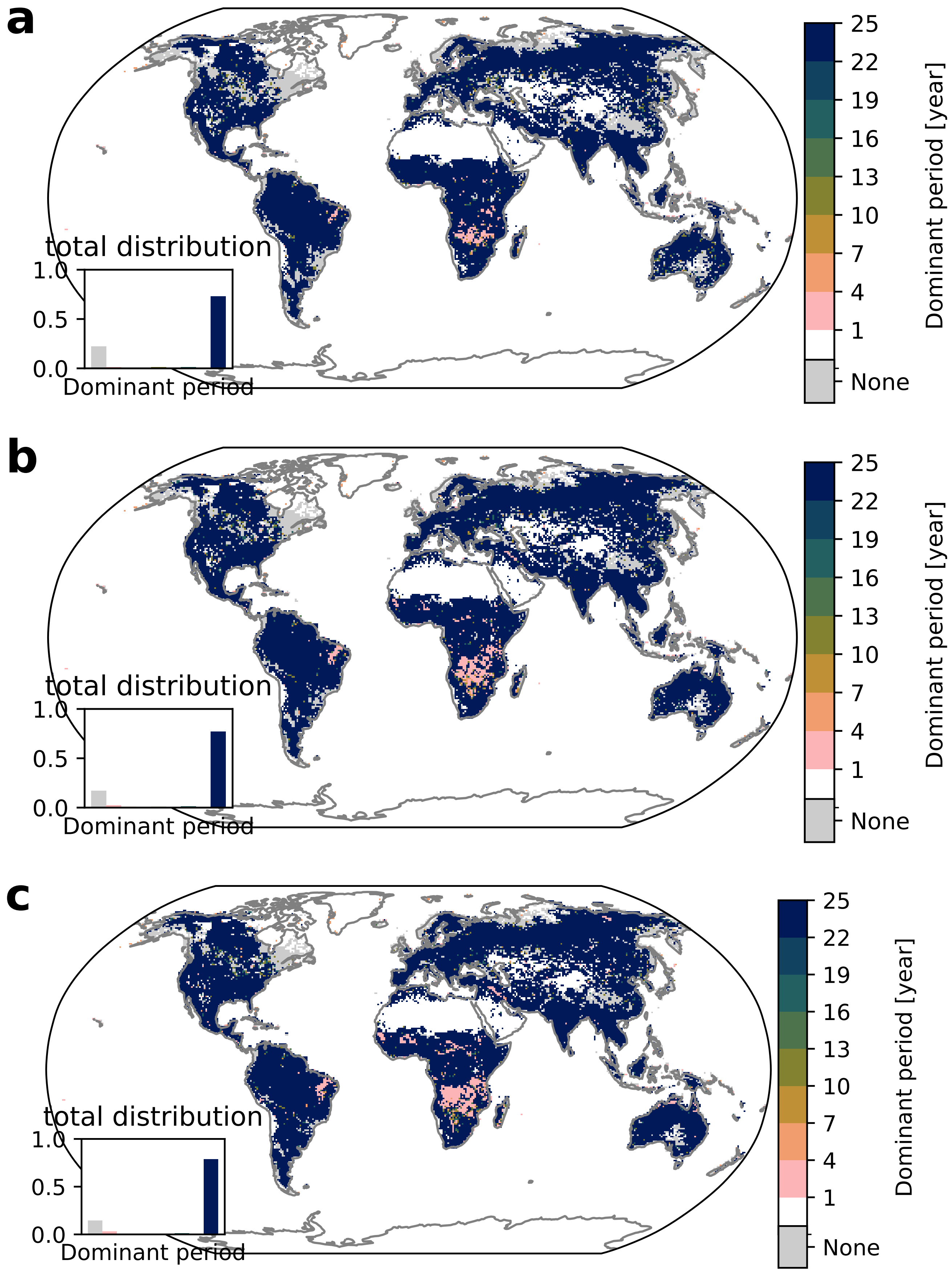

Supplementary Figure 12: **Dominant periods for wildfire caps II.** Median dominant period of wildfires for a wildfire cap of (**a**) 20%, (**b**) 10%, and (**c**) 5%. The gray color signifies no dominant period (irregularity) while existing dominant periods are grouped in three-year intervals ranging from 1-4 years (pink) to 22-25 years (blue). The inset shows the distribution of the dominant period counts.

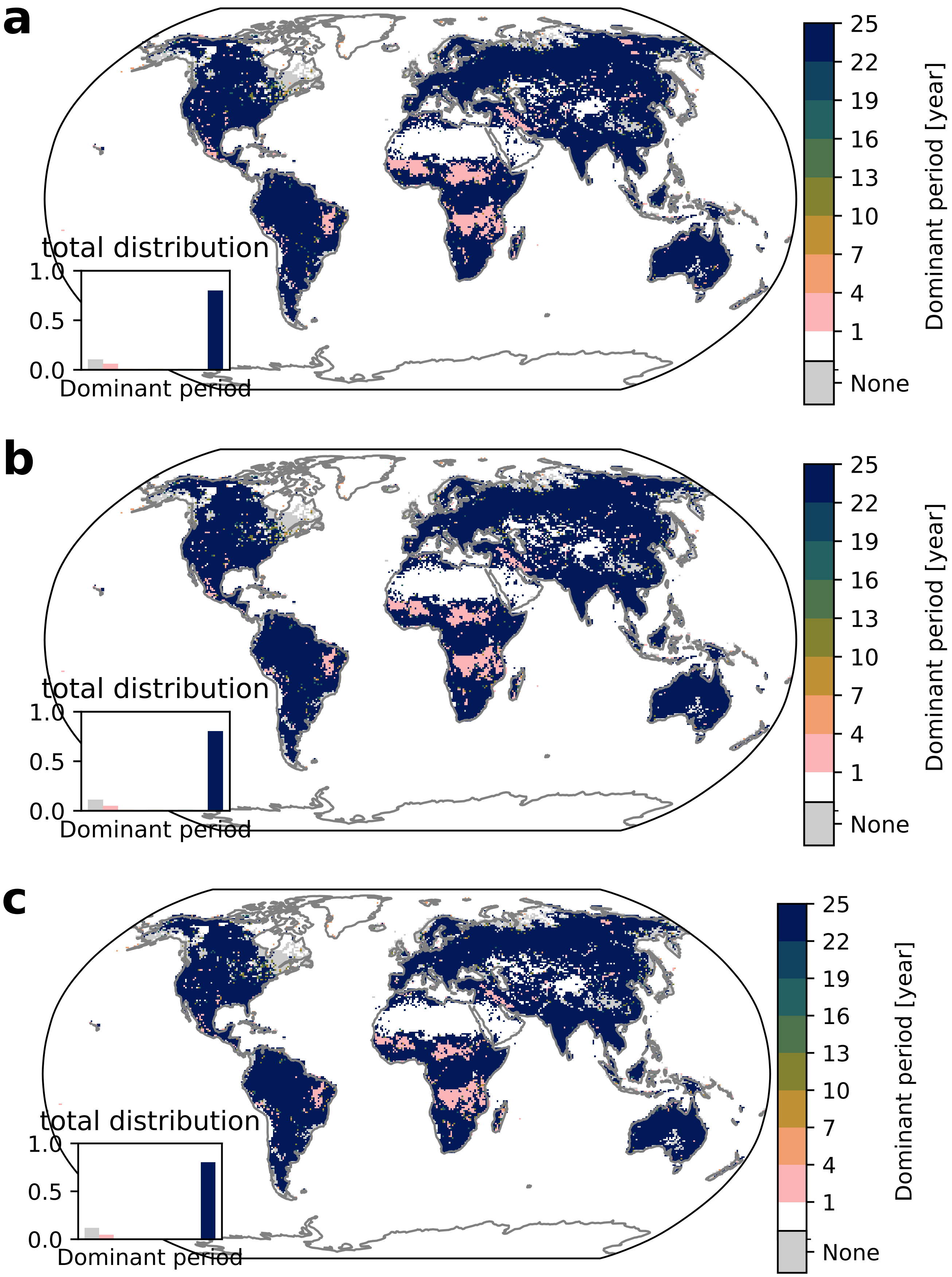

Supplementary Figure 13: **Dominant periods for wildfire caps III.** Median dominant period of wildfires for a wildfire cap of (**a**) 1%, (**b**) 1.5%, and (**c**) 2%. The gray color signifies no dominant period (irregularity) while existing dominant periods are grouped in three-year intervals ranging from 1-4 years (pink) to 22-25 years (blue). The inset shows the distribution of the dominant period counts.

6 Supplementary Discussion: Regional event counts

To estimate the extension of extreme event impacted areas we count the modeled occurrences within each time window. Mathematically speaking we define

$$N^{jk}_{it_0\Delta T}(\theta,\phi) = \sum_{m=0}^{2\Delta T-1} \mathbb{I}_{(0,\infty)}(f^{jk}_i(t_0+m,\theta,\phi)), \tag{7}$$

where $\mathbb{I}$ is the indicator function. The results for the median crop failure under historical and SSP5-8.5 climate conditions and for time windows 1850-1899, 1950-1999 and 2050-2099 are shown in Supplementary Fig. 14.

We observe a strong increase in counts at low latitudes while in the past we observed a more homogeneous distribution of extreme events with crop failures never exceeding 30 occurrences in a 50 year time window. On the other hand, in the future time window 2050-2099 we find several world regions where we have yearly crop failure according to our definition. This strong increase in affected area counts is responsible for the strong trend in the dominant period (see Sec. 2 in the main text) where we mostly find only largest dominant periods (quasi-regular increase in affected area) or irregularity (irregular increase in affected area).

The results for heatwaves are shown in Supplementary Fig. 15.
Here, we observe very extreme event counts in 1850-1899 as extreme temperature conditions are seldom met. Drastic changes are seen in the future where large counts are predicted at low latitudes.

A different situation is observed for wildfire (see Supplementary Fig. 16), where we observe future strong increases in counts in the Americas and Eurasia while the transition phase 1950-1999 shows only minor changes compared to the other impact categories.
This perspective is consistent with the relative small increase in total affected area (see Fig. 2 in the main document). A clearer picture on the dynamics is obtained when looking at the dominant period (see Sec. 2 in the main text).

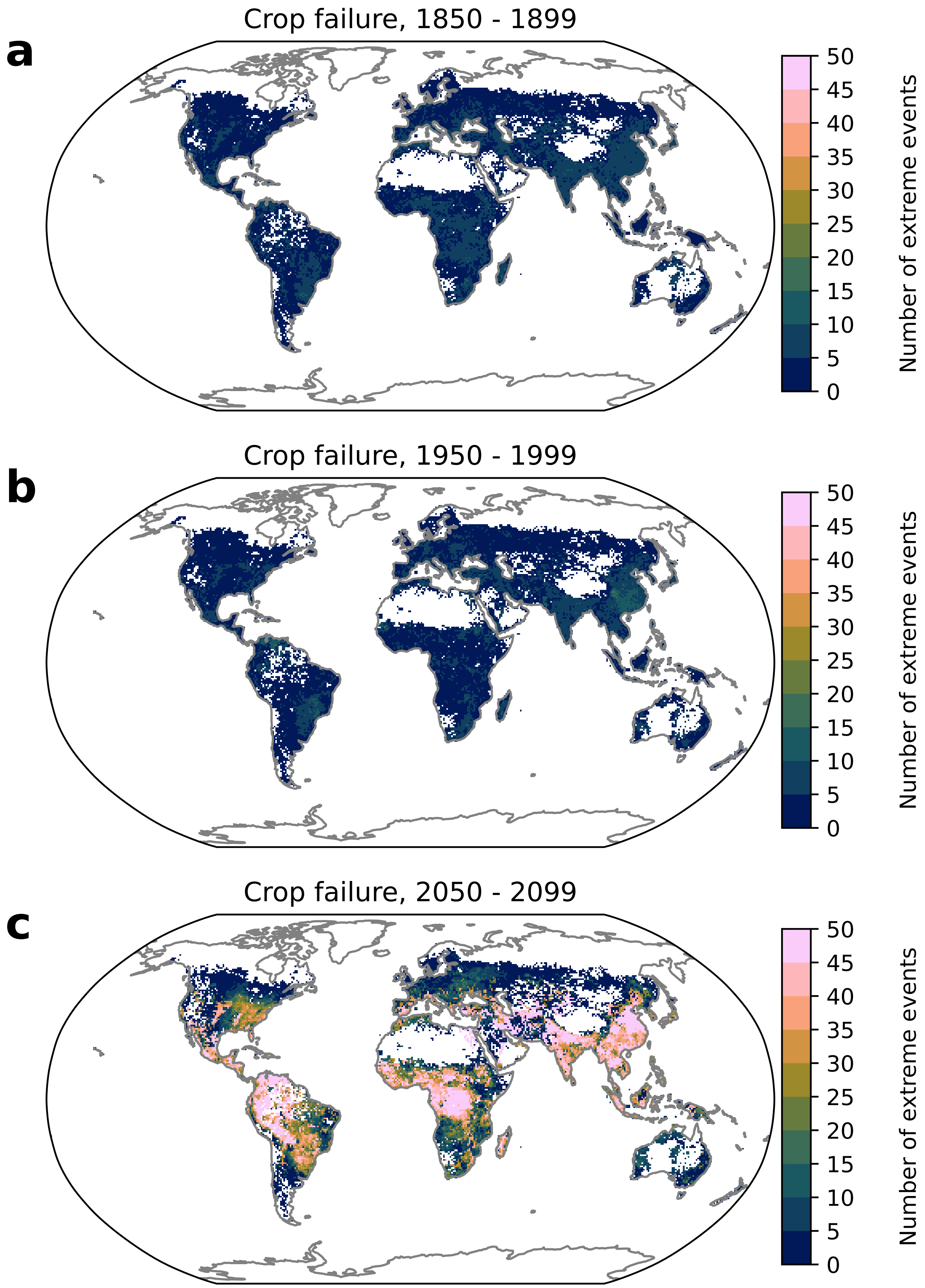

Supplementary Figure 14: **Number of crop failures.** Median count of crop failure in (**a**) 1850-1899, (**b**) 1950-1999, and (**c**) 2050-2099 under SSP5-8.5.

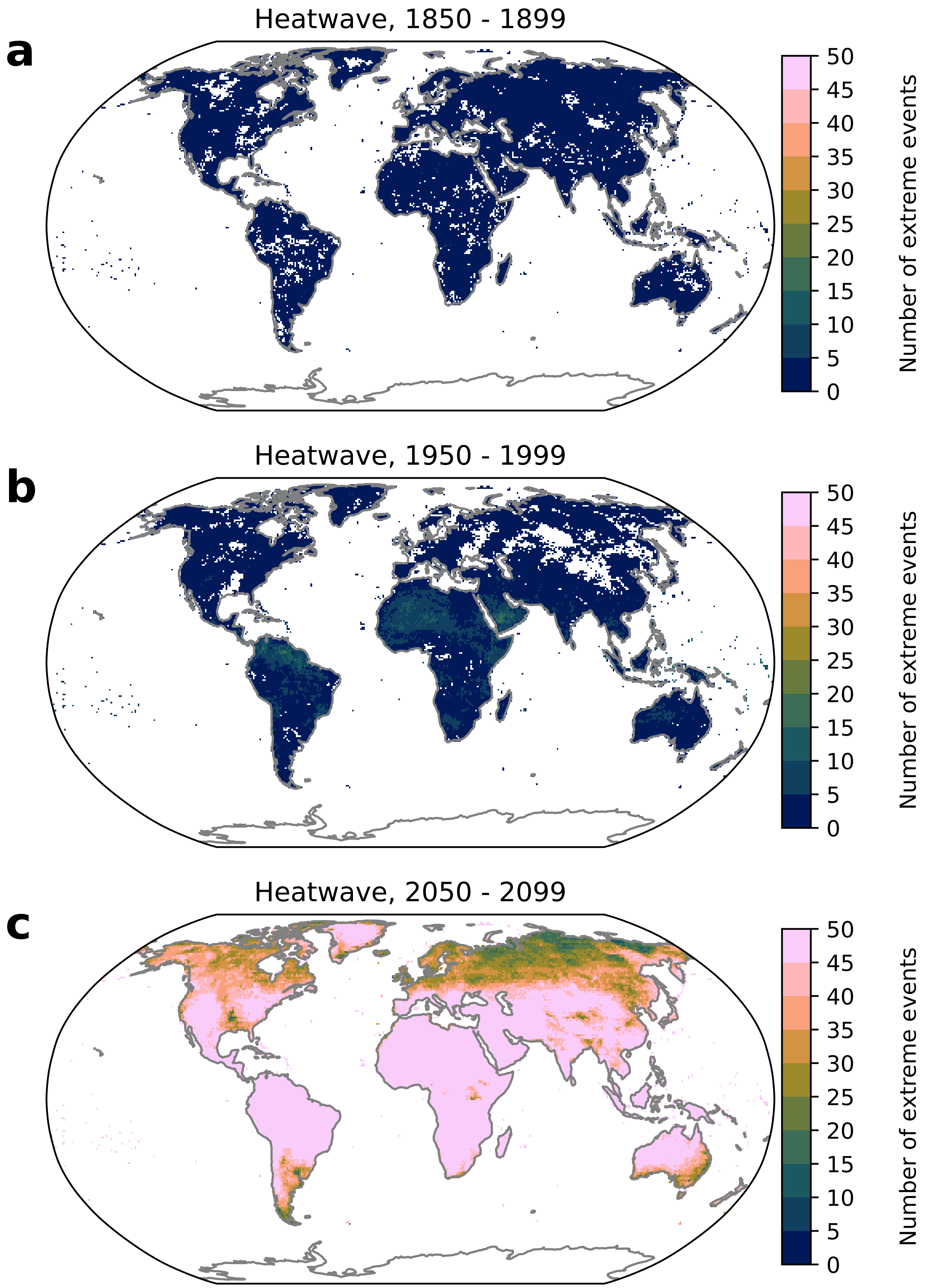

Supplementary Figure 15: **Number of heatwaves.** Median count of heatwaves in (**a**) 1850-1899, (**b**) 1950-1999, and (**c**) 2050-2099 under SSP5-8.5.

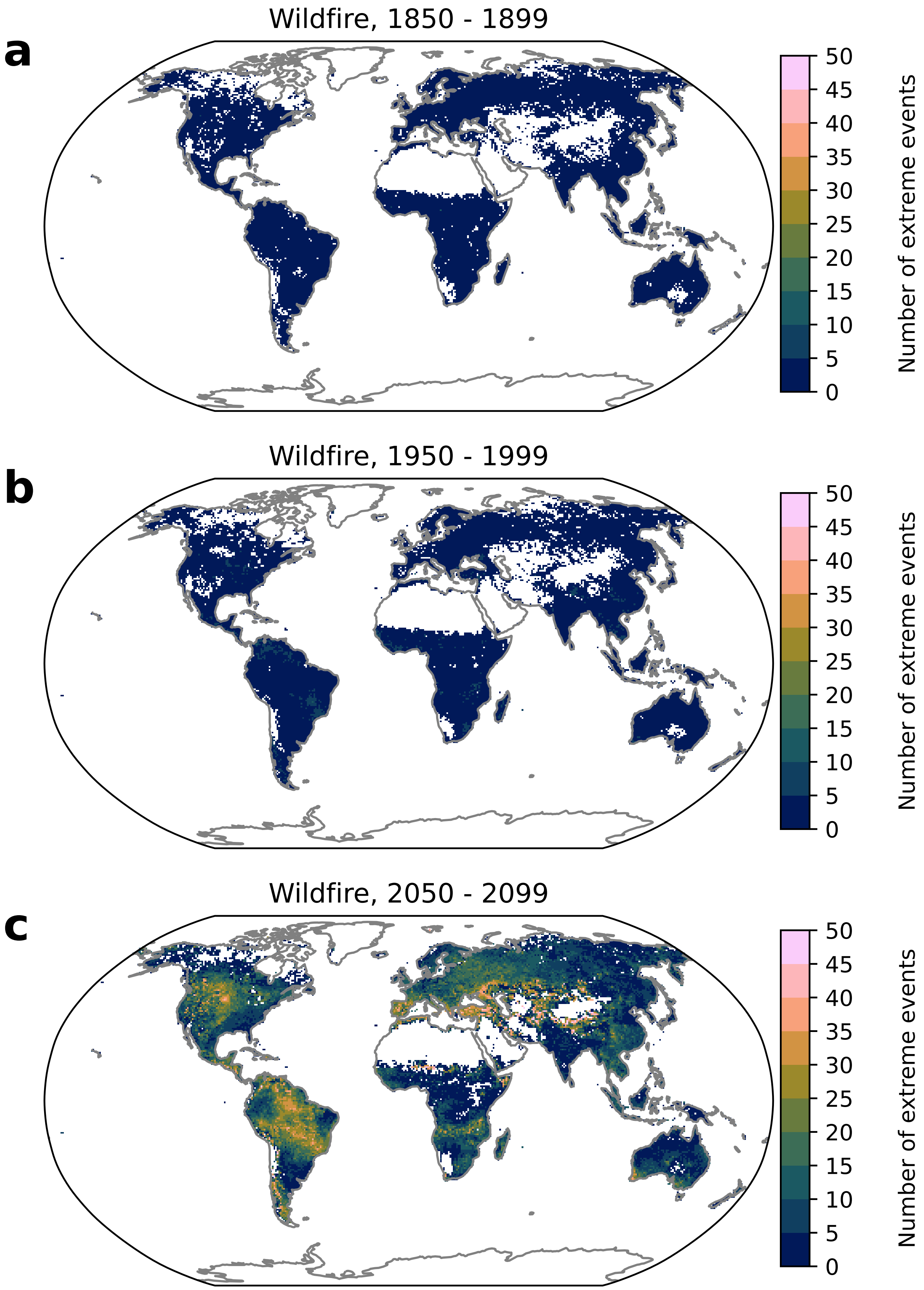

Supplementary Figure 16: **Number of wildfires.** Median count of wildfire affected area in (**a**) 1850-1899, (**b**) 1950-1999, and (**c**) 2050-2099 under SSP5-8.5.

7 Supplementary Discussion: Results for SSP1-2.6 and SSP3-7.0

We present the dominant period results for SSP1-2.6 and SSP3-7.0 in the time interval 2040-2069 for all impact types, namely crop failure, heatwave, and wildfire.

7.1 Crop failure

Within the SSP1-2.6 scenario we find a consistent global distribution of regions exhibiting regularity (see Supplementary Fig. 17) compared to the SSP5-8.5 results (see Fig. 3 b and c in the main text).

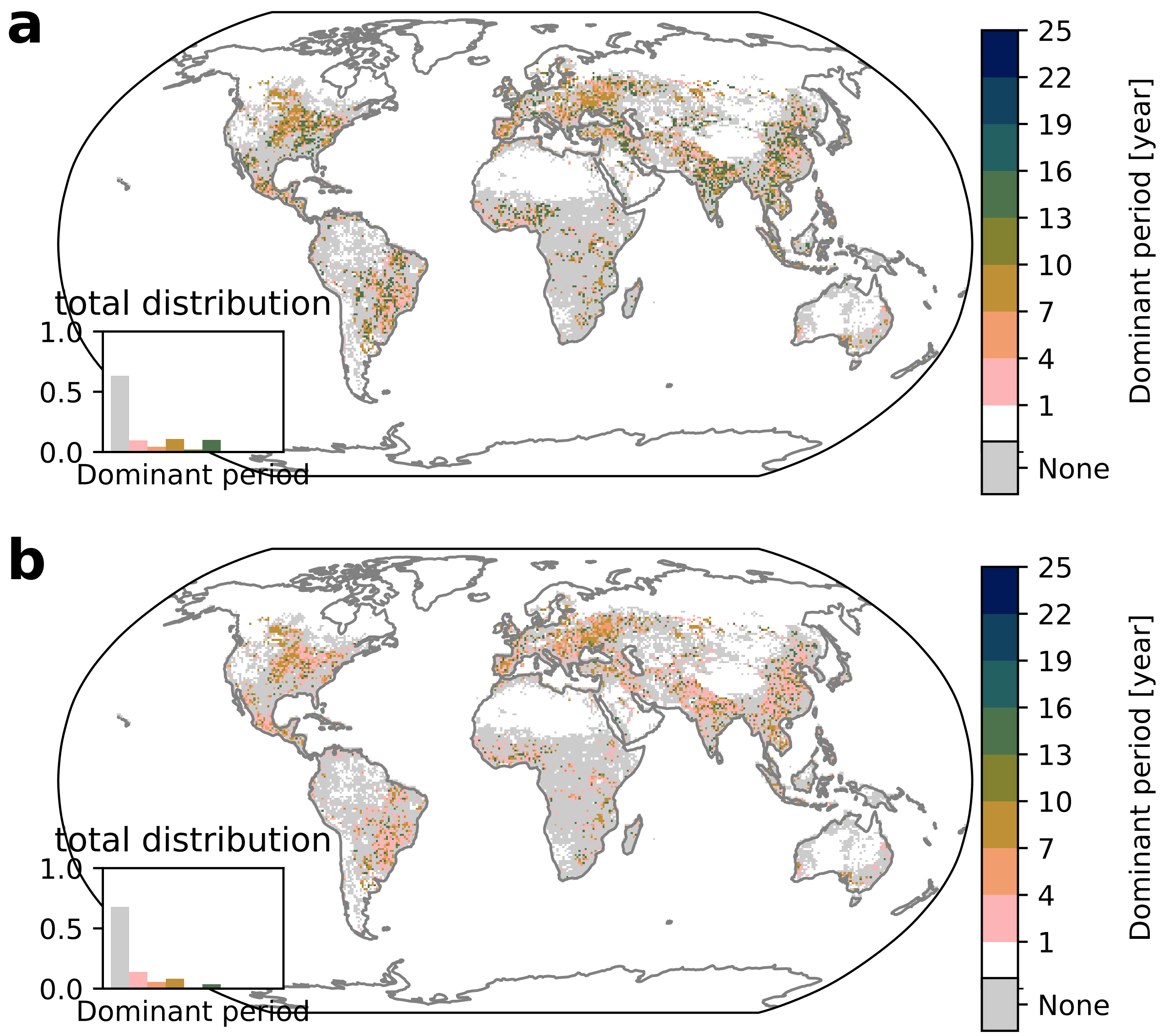

Supplementary Figure 17: **Crop failure dominant periods for SSP1-2.6.** Median dominant period of crop failures for SSP1-2.6 (**a**) for the period 2040-2069 and (**b**) the respective linearly detrended results. The gray color signifies no dominant period (irregularity) while existing dominant periods are grouped in three-year intervals ranging from 1-4 years (pink) to 22-25 years (blue). The inset shows the distribution of the dominant period counts.

The main difference between the two SSP results are the larger sections of largest dominant period (10% in SSP1-2.6 and 25% in SSP5-8.5) due to the stronger warming trend. Dominant periods between 7 and 13 years that were previously observed in picontrol (see Fig. 1 in the main text) are mostly retained in the northern hemisphere where extreme precipitation and heat events are less.

Through linear detrending we are again able to partially remove the warming trend as expressed in largest dominant periods (see Supplementary Fig. 17 b). Similarly to the SSP5-8.5 result we observe the emergence of highest dominant periods in the detrended results.

The results for SSP3-7.0 are even closer to the SSP5-8.5 results (see Supplementary Fig. 18) both for the original and the linear detrended results. In the case of the original results we observe

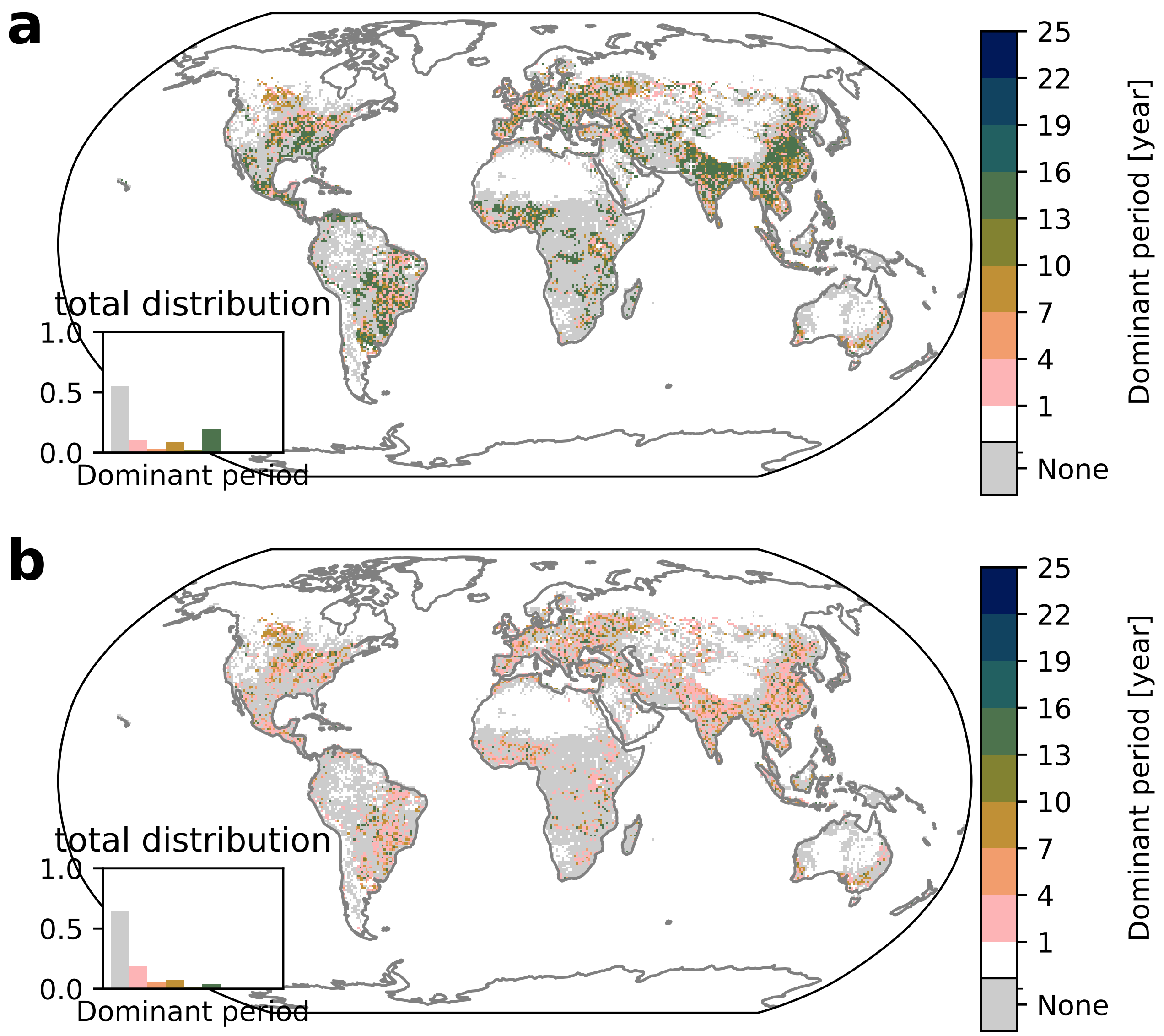

Supplementary Figure 18: **Crop failure dominant periods for SSP1-3.70.** Median dominant period of crop failures for SSP3-7.0 (**a**) for the period 2040-2069 and (**b**) the respective linearly detrended results. The gray color signifies no dominant period (irregularity) while existing dominant periods are grouped in three-year intervals ranging from 1-4 years (pink) to 22-25 years (blue). The inset shows the distribution of the dominant period counts.

a prevalence of irregularity and largest dominant periods. This is clear sign of the strong warming trend which can be partly absorbed through linear detrending where the highest dominant frequencies emerge and even stronger so compared to SSP1-2.6.

7.2 Heatwave

For heatwaves we observe a weaker warming trend as expressed through the occurrence of largest dominant periods (compare Supplementary Fig. 19 (a) to Fig. 4 (b) in the main text).

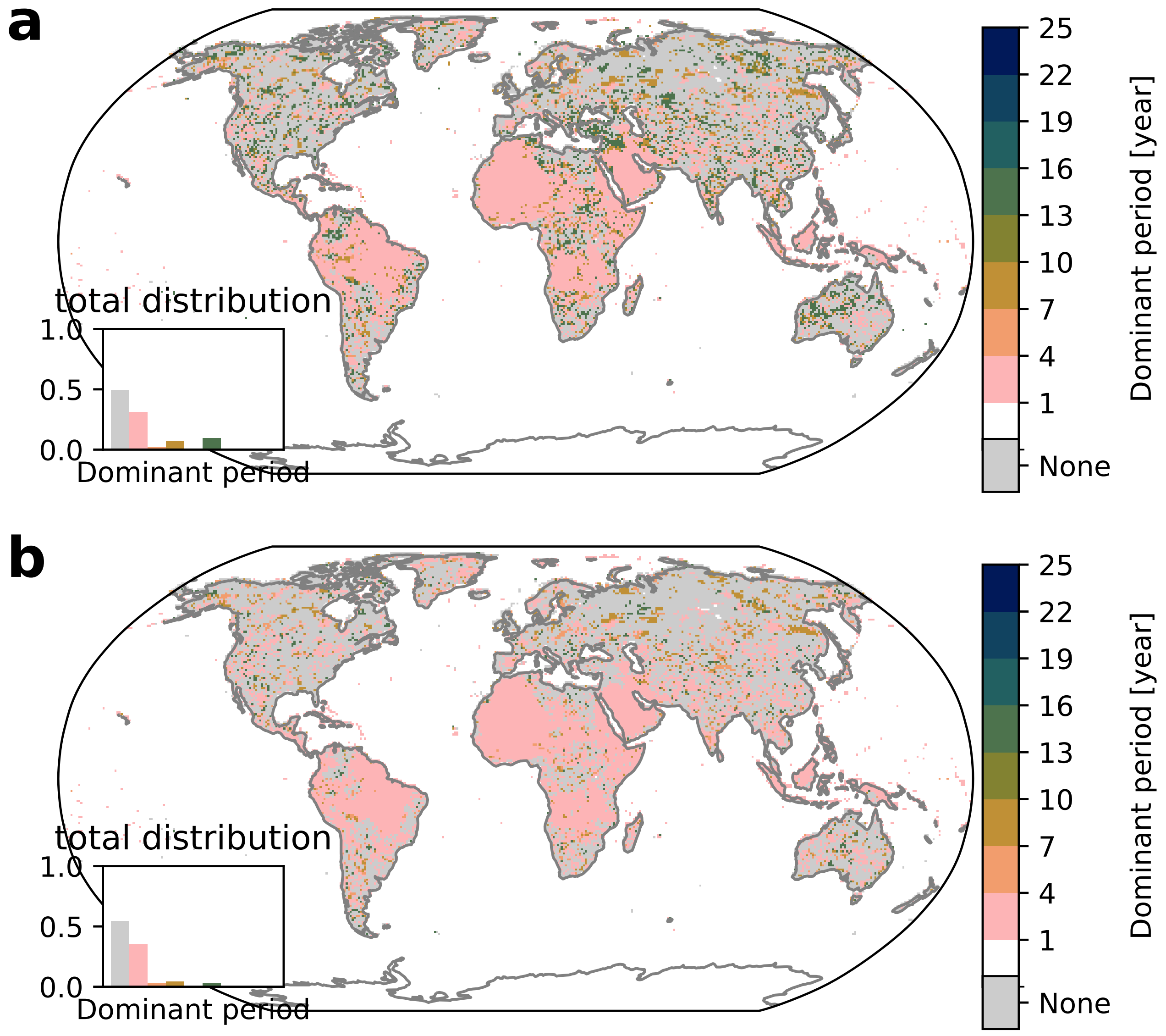

Supplementary Figure 19: **Heatwave dominant periods for SSP1-2.6.** Median dominant period of heatwaves for SSP1-2.6 (**a**) for the period 2040-2069 and (**b**) the respective linearly detrended results. The gray color signifies no dominant period (irregularity) while existing dominant periods are grouped in three-year intervals ranging from 1-4 years (pink) to 22-25 years (blue). The inset shows the distribution of the dominant period counts.

As observed in the SSP5-8.5 case linear detrending leads to a shift towards lowest dominant periods (see Supplementary Fig. 19 b). Note that the shift towards smallest dominant periods is smaller than in the SSP5-8.5 due to the relatively smaller global warming (see Fig. 4 c in the main text).

The results for SSP3-7.0 are already very close to the SSP5-8.5 results (see Supplementary Fig. 20) in terms of global distribution of regularity and irregularity regions. For example, within the linearly detrended results we observe mostly smallest dominant periods or irregularity for both scenarios.

7.3 Wildfire

In the case of wildfires we observe a similar distribution between SSP1-2.6 and SSP5-8.5 results (compare Supplementary Fig. 21 (a) to Fig. 5 (b) and (c) in the main text) with fewer smallest dominant periods in the case of SSP1-2.6.

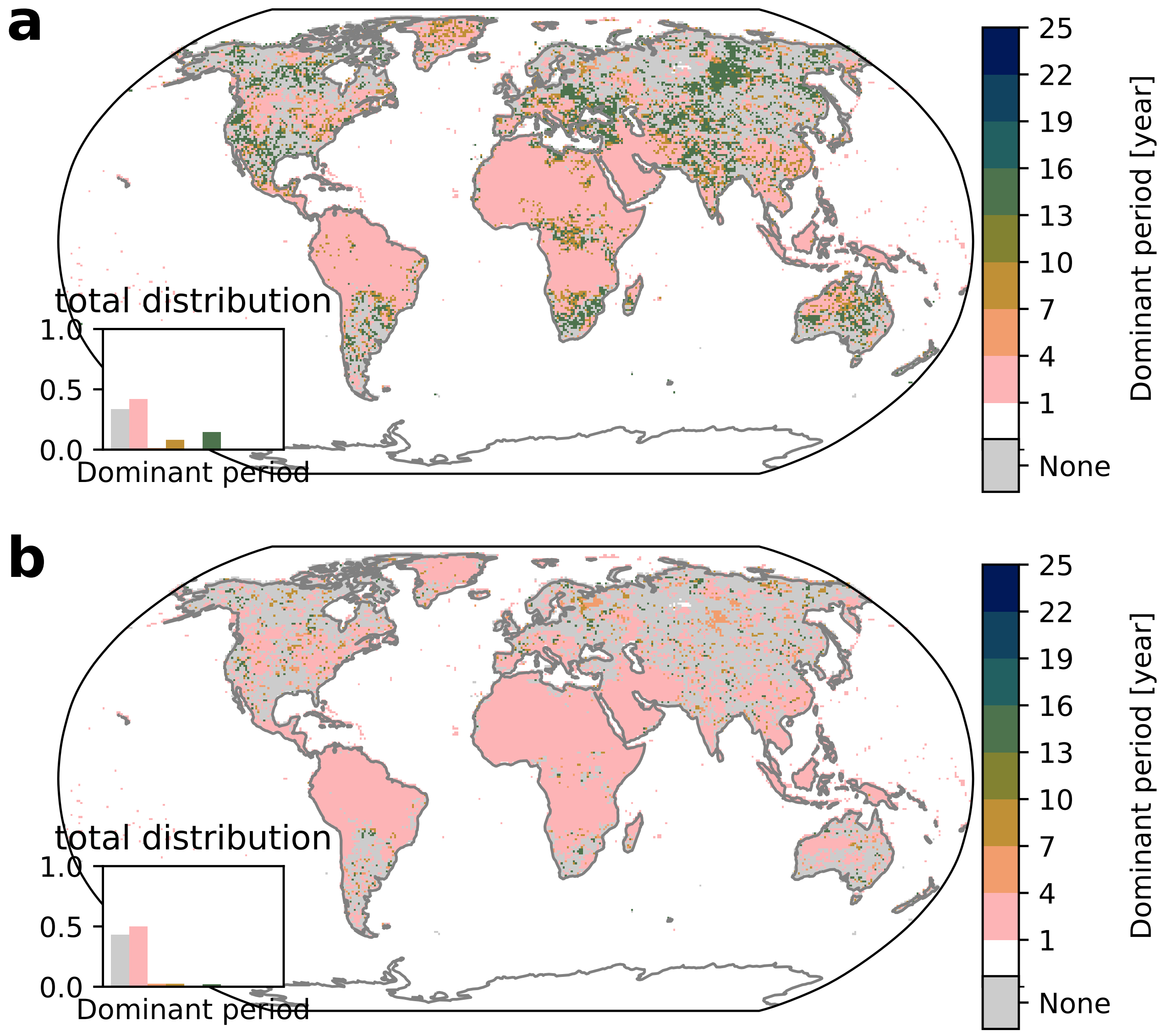

Supplementary Figure 20: **Heatwave dominant periods for SSP3-7.0.** Median dominant period of heatwaves for SSP3-7.0 (**a**) for the period 2040-2069 and (**b**) the respective linearly detrended results. The gray color signifies no dominant period (irregularity) while existing dominant periods are grouped in three-year intervals ranging from 1-4 years (pink) to 22-25 years (blue). The inset shows the distribution of the dominant period counts.

Even in the case of SSP3-7.0 we mainly observe an extension of highest dominant period regions in both Americas and Asia (see Supplementary Fig. 21 b).

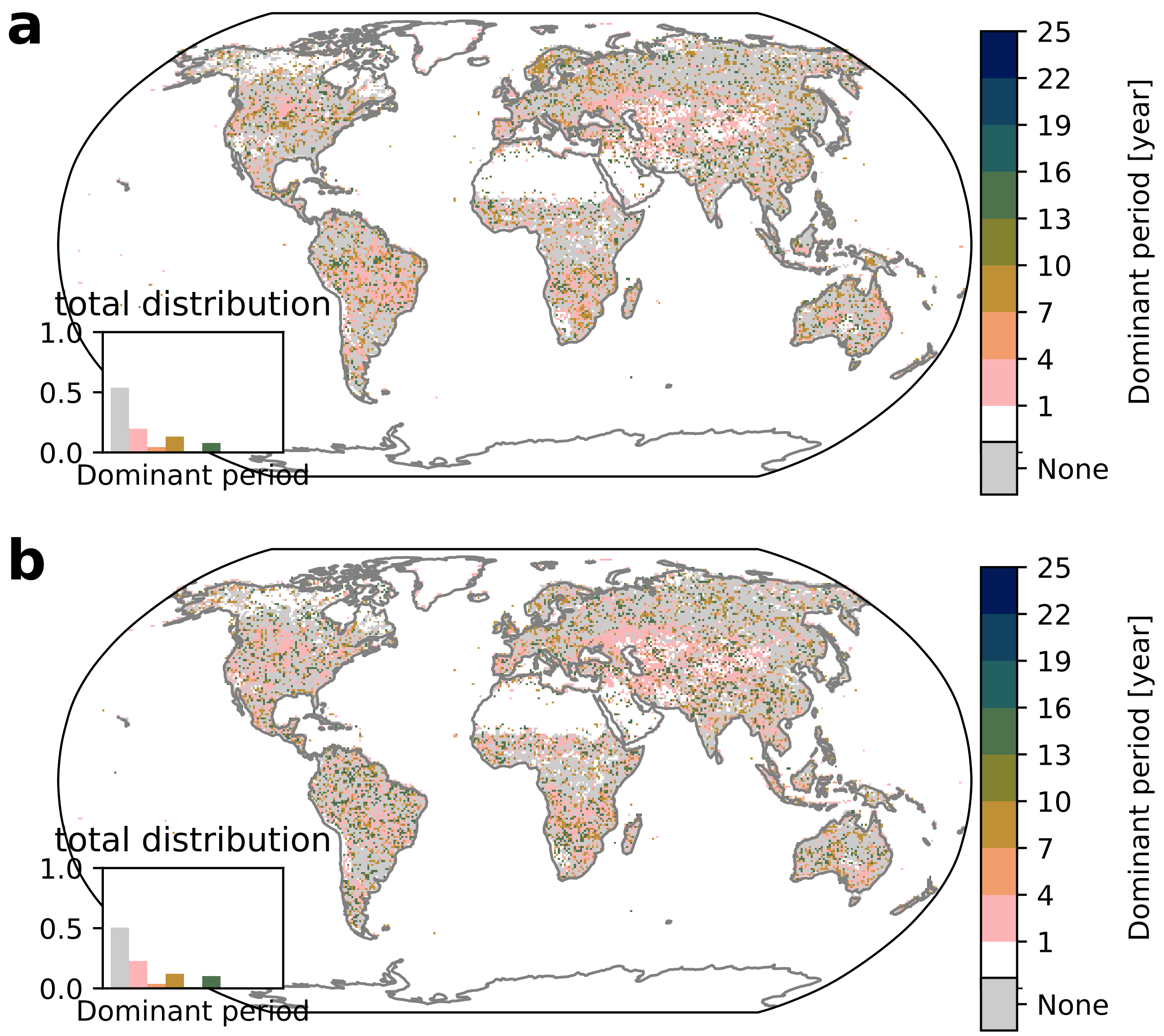

Supplementary Figure 21: **Wildfire dominant periods for SSP1-2.6 and SSP3-7.0.** Median dominant period of wildfires for the period 2040-2069 (**a**) for SSP1-2.6 and (**b**) SSP3-7.0. The gray color signifies no dominant period (irregularity) while existing dominant periods are grouped in three-year intervals ranging from 1-4 years (pink) to 22-25 years (blue). The inset shows the distribution of the dominant period counts.

8 Supplementary Discussion: Number of contributing models

In order to estimate the significance of our dominant periods in the SSP5-8.5 scenario we count the number of models that find a dominant period at each grid cell for crop failure (Supplementary Fig. 22), heatwaves (Supplementary Fig. 23), and wildfire (Supplementary Fig. 24). All impact types show the same consistent behavior: At $t_0 = 1950$ where we are at the transitioning point between pre-industrial climate conditions and on-setting extreme event impact increase we find a stark increase of irregularity which is also reflected in the small number of models contributing to the remaining regularity signals.
When moving to $t_0 = 2040$ we leave the transition point behind and find a more regular warming trend reflected in increased regularity and large dominant periods (see Sec. 2 in the main text). Analogously, we observe more models to contribute to the regularity signal.
Through linear detrending we are able to partially account for the warming trend which reveals not only a shift towards higher frequencies but also more irregularity as discussed in the main document. This shift towards higher frequencies and more irregularity is reflected in the reduced number of models contributing to the regularity signal.

In the case of the picontrol runs we present the restricted median model counts in analogy to the median dominant period (see Sec. 4). We observe fewer contributing models on the geographical scale due to the percentile definition of extreme events (see Supplementary Fig. 25).

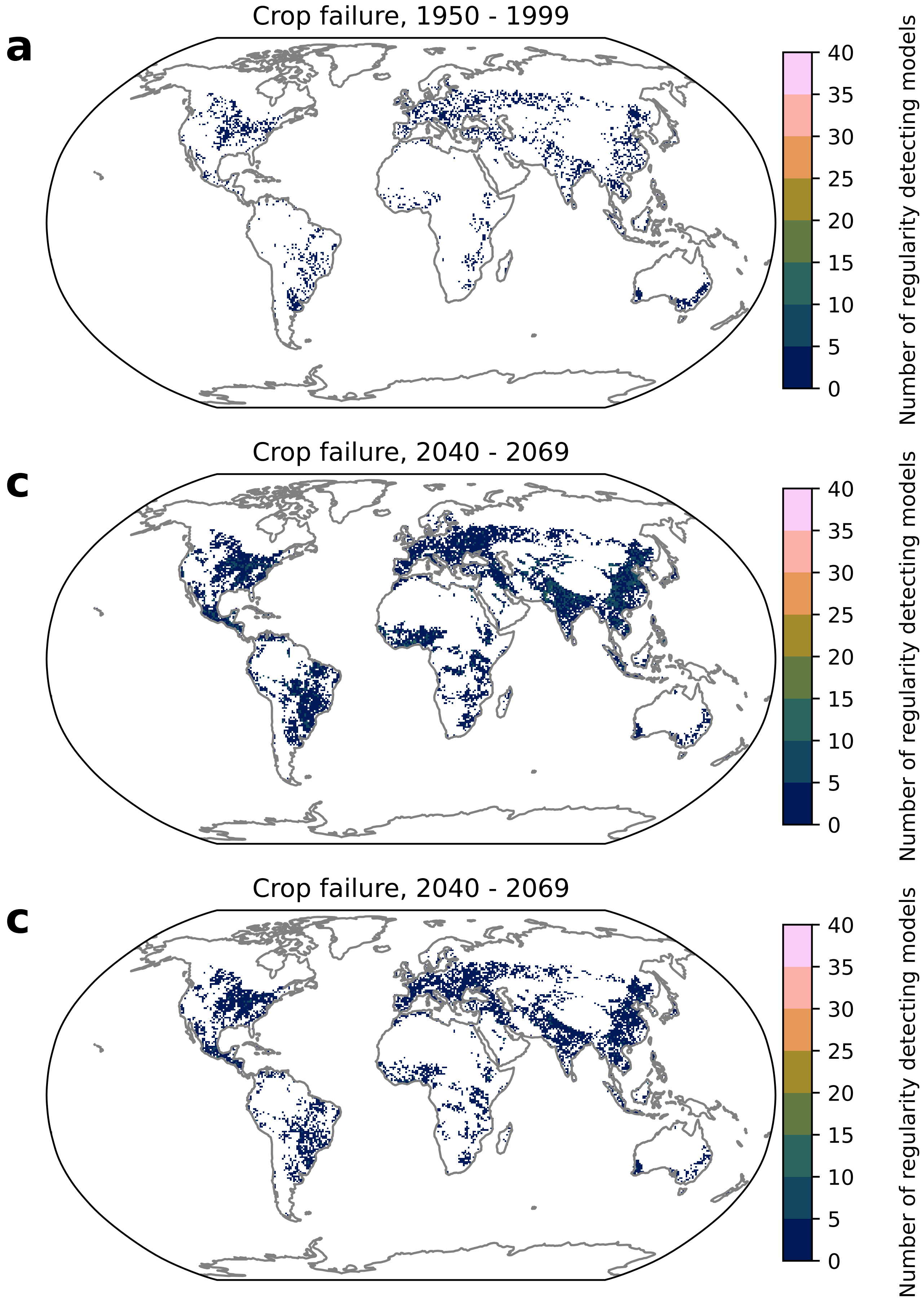

Supplementary Figure 22: **Number of detecting models for crop failure.** Number of model combinations that detect a dominant period in SSP5-8.5 for crop failure for (**a**) 1950-1999, (**b**) 2040-2069, and (**c**) detrended 2040-2069.

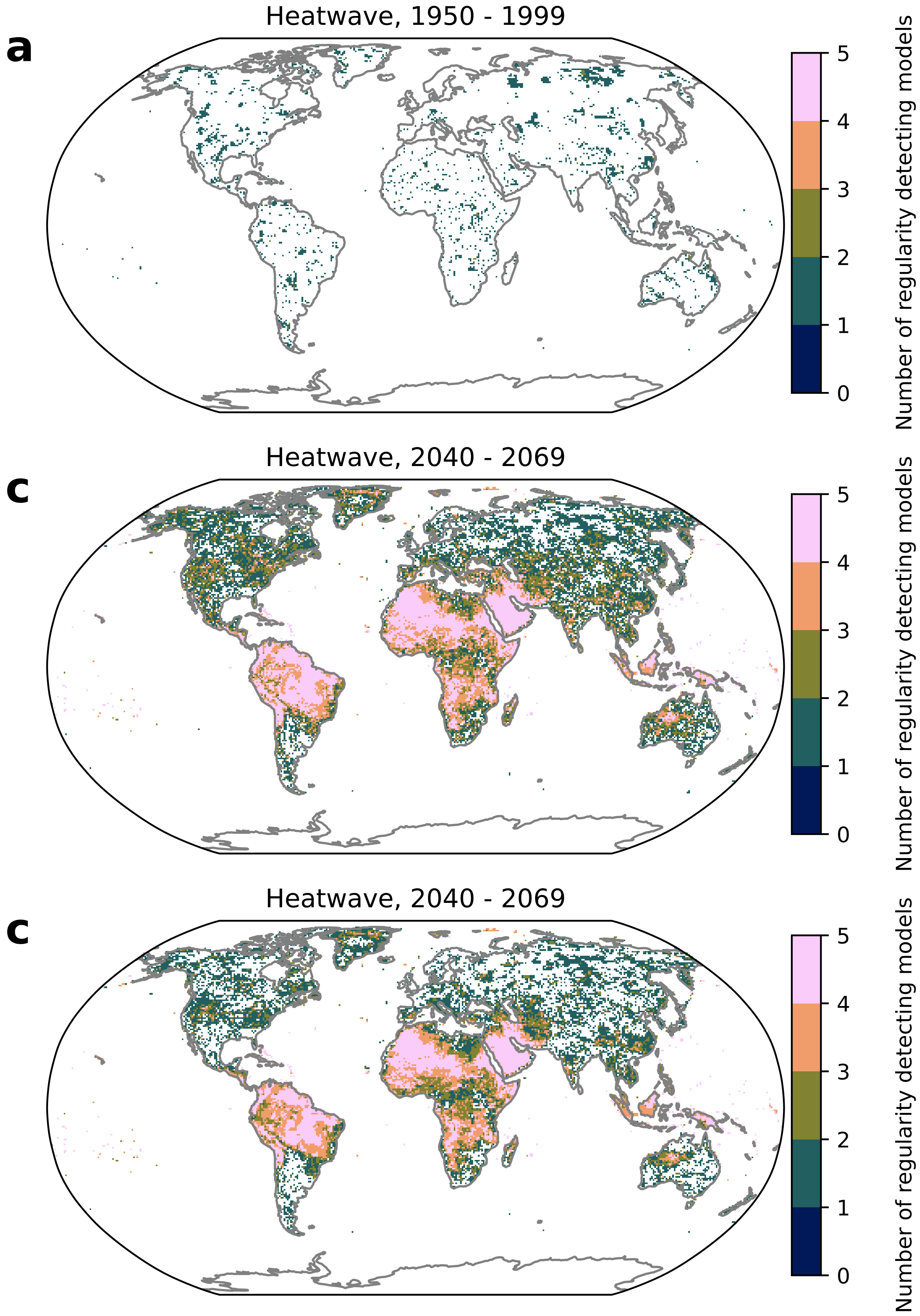

Supplementary Figure 23: **Number of detecting models for heatwave.** Number of model combinations that detect a dominant period in SSP5-8.5 for heatwave for (**a**) 1950-1999, (**b**) 2040-2069, and (**c**) linearly detrended 2040-2069.

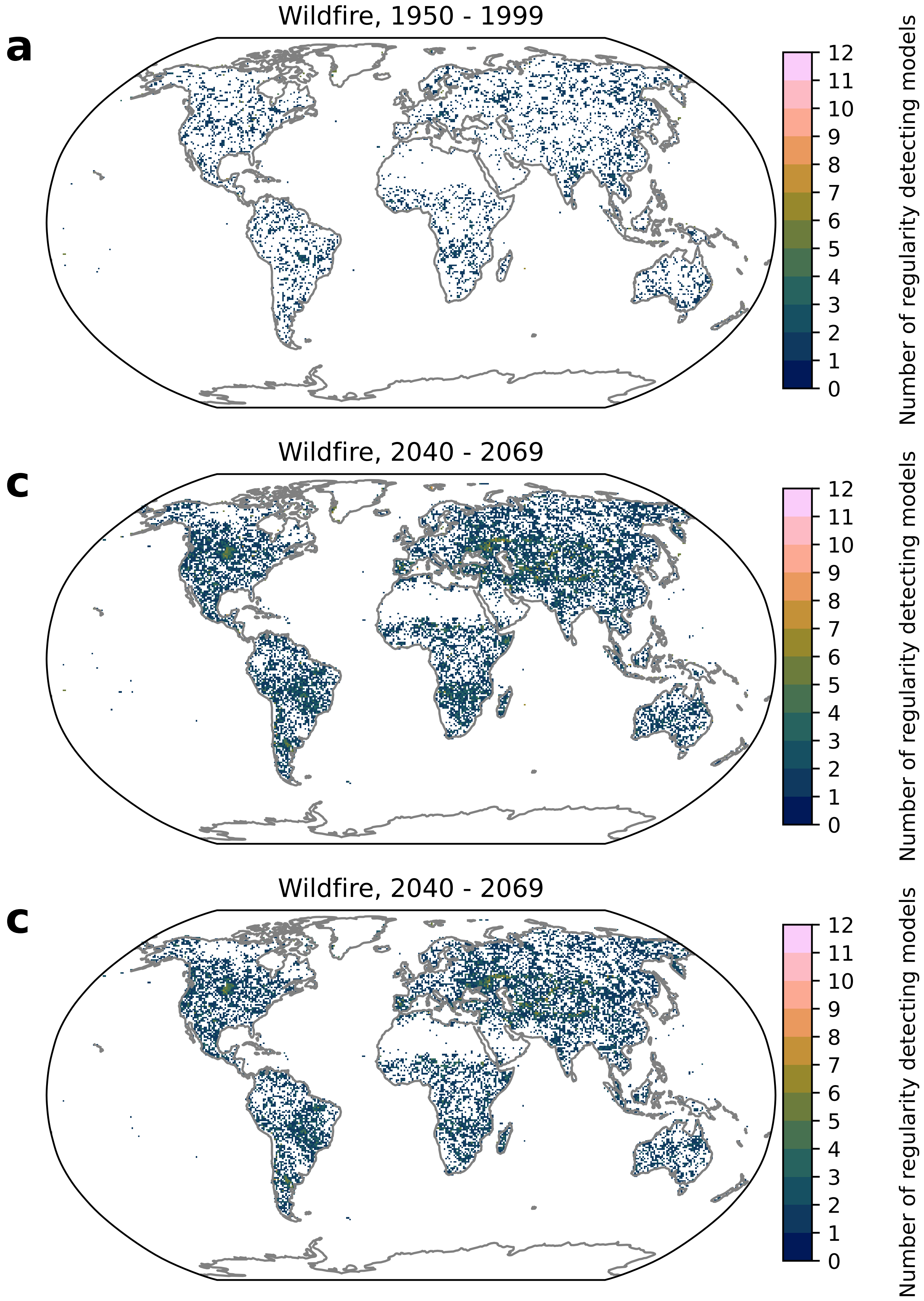

Supplementary Figure 24: **Number of detecting models for wildfire.** Number of model combinations that detect a dominant period in SSP5-8.5 for wildfire for (**a**) 1950-1999, (**b**) 2040-2069, and (**c**) linearly detrended 2040-2069.

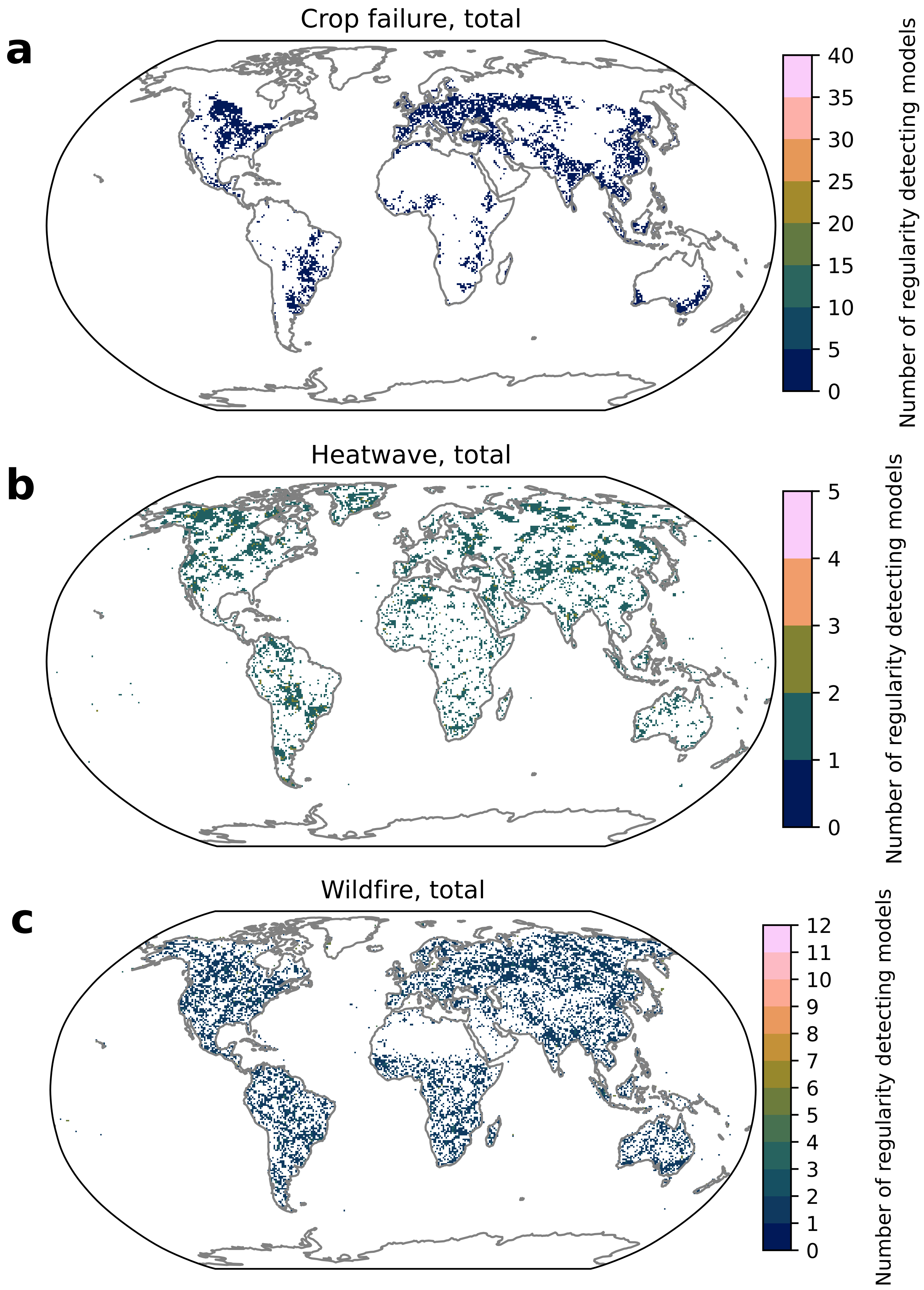

Supplementary Figure 25: **Number of detecting models for picontrol.** Number of model combinations that detect a dominant period in picontrol for (**a**) crop failure, (**b**) heatwave, and (**c**) wildfire in the time windows 1850-1899, 1900-1949, …, 2050-2099.

9 Supplementary Discussion: SSP5-8.5 results for different time windos

In this section we show dominant period results for different time windows and time window sizes $2\Delta T$ at the end of century within the SSP5-8.5 scenario.

9.1 Dominant periods for 2070-2099

In the case of $2\Delta = 30$ years for the time window 2070-2099 we find a continued warming trend for crop failure (see Supplementary Fig. 26) in comparison to the results in the main text (see Fig. 3 (b), (c) in the main text).

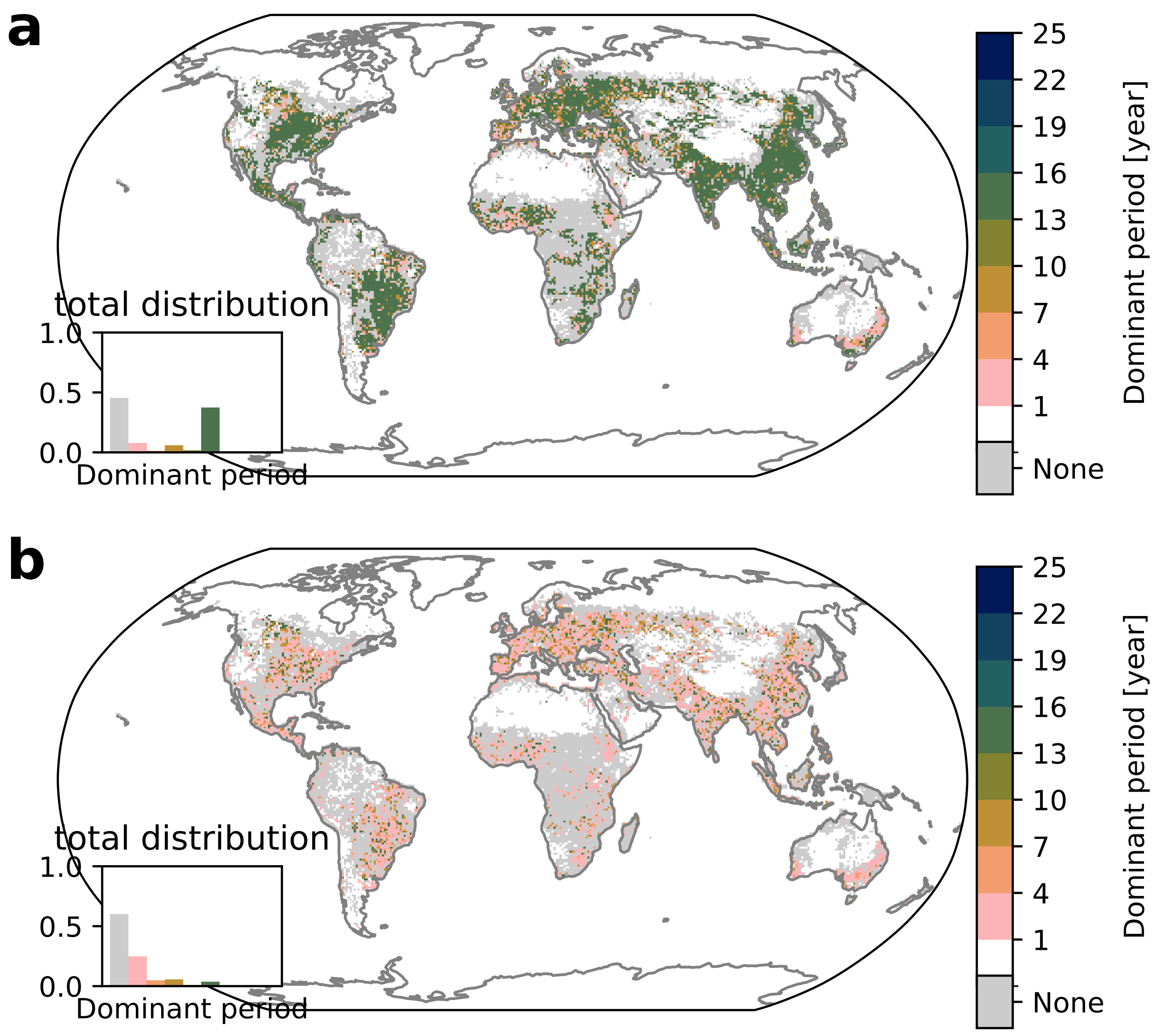

Supplementary Figure 26: **Dominant period for crop failure in 2070-2099.** Median dominant period for crop failure and (**a**) 2070-2099, and (**b**) linearly detrended 2070-2099 under SSP5-8.5. The white color signifies no extreme climate impact occurrence and gray color signifies no dominant period (irregularity) while existing dominant periods are grouped in three-year intervals ranging from 1-4 years to 13-16 years (blue). The inset shows the distribution of the dominant period counts.

Similarly, in the case of heatwaves we find an even stronger warming effect in 2070-2099 compared to 2040-2069 (see Supplementary Fig. 27). At the end of century we find that all almost all

world areas are affected by heat compared to the picontrol reference.

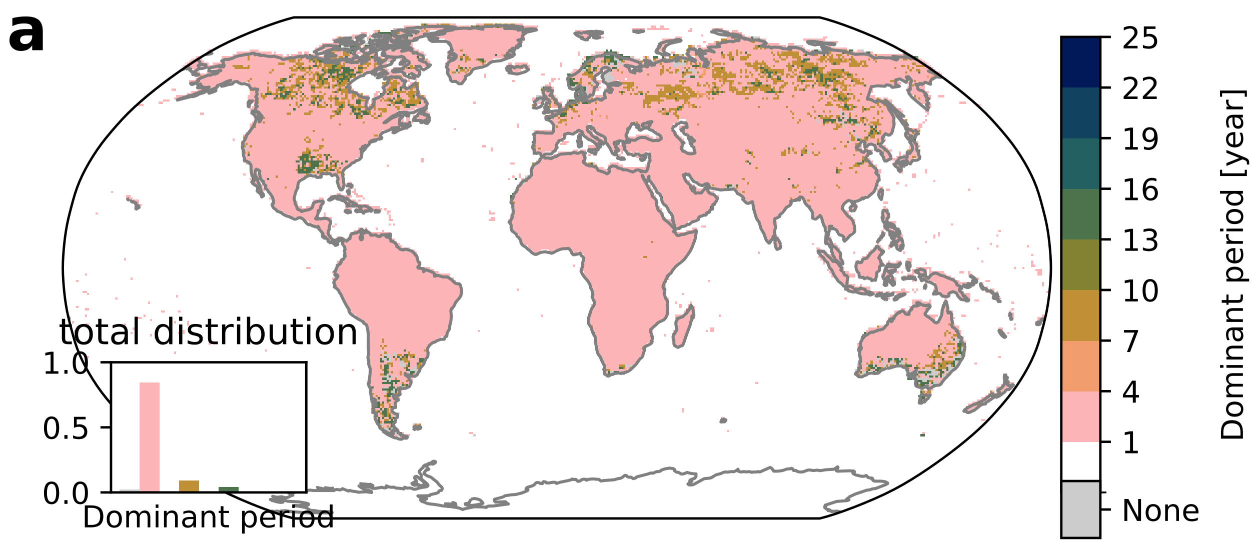

Supplementary Figure 27: **Dominant period for heatwave in 2070-2099.** Median dominant period for heatwaves and (**a**) 2070-2099, and (**b**) linearly detrended 2070-2099 under SSP5-8.5. The white color signifies no extreme climate impact occurrence and gray color signifies no dominant period (irregularity) while existing dominant periods are grouped in three-year intervals ranging from 1-4 years to 13-16 years (blue). The inset shows the distribution of the dominant period counts.

In the case of wildfires we find a smaller change of dominant period distribution in 2070-2099 compared to the 2040-2069 time frame (see Supplementary Fig. 28).

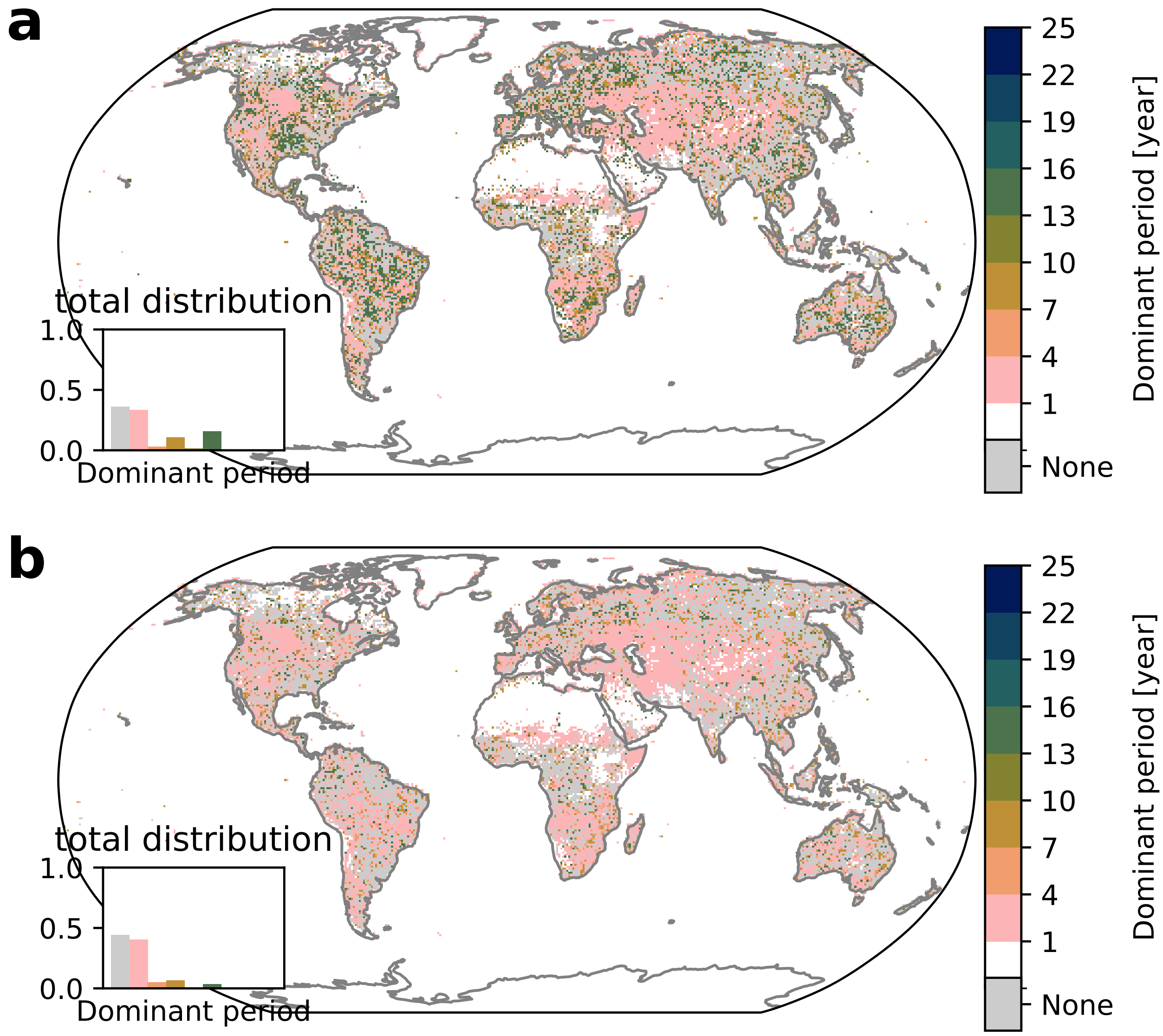

Supplementary Figure 28: **Dominant period for wildfire in 2070-2099.** Median dominant period for wildfire and (**a**) 2070-2099, and (**b**) linearly detrended 2070-2099 under SSP5-8.5. The white color signifies no extreme climate impact occurrence and gray color signifies no dominant period (irregularity) while existing dominant periods are grouped in three-year intervals ranging from 1-4 years to 13-16 years (blue). The inset shows the distribution of the dominant period counts.

9.2 Dominant periods for 2050-2099

Here, we consider the time windows 2050-2099 which corresponds to $2\Delta T = 50$y. In the case of crop failure we mainly observe irregularity or a strong trend as signified by the dominant period 22-25 years (see Supplementary Fig. 29 a). Through linear detrending we are able to partially absorb the superlinear trend which results in more irregularity but also a minor increase in smallest dominant periods consistent with the results in the main text (see Fig. 3 b and c in the main text).

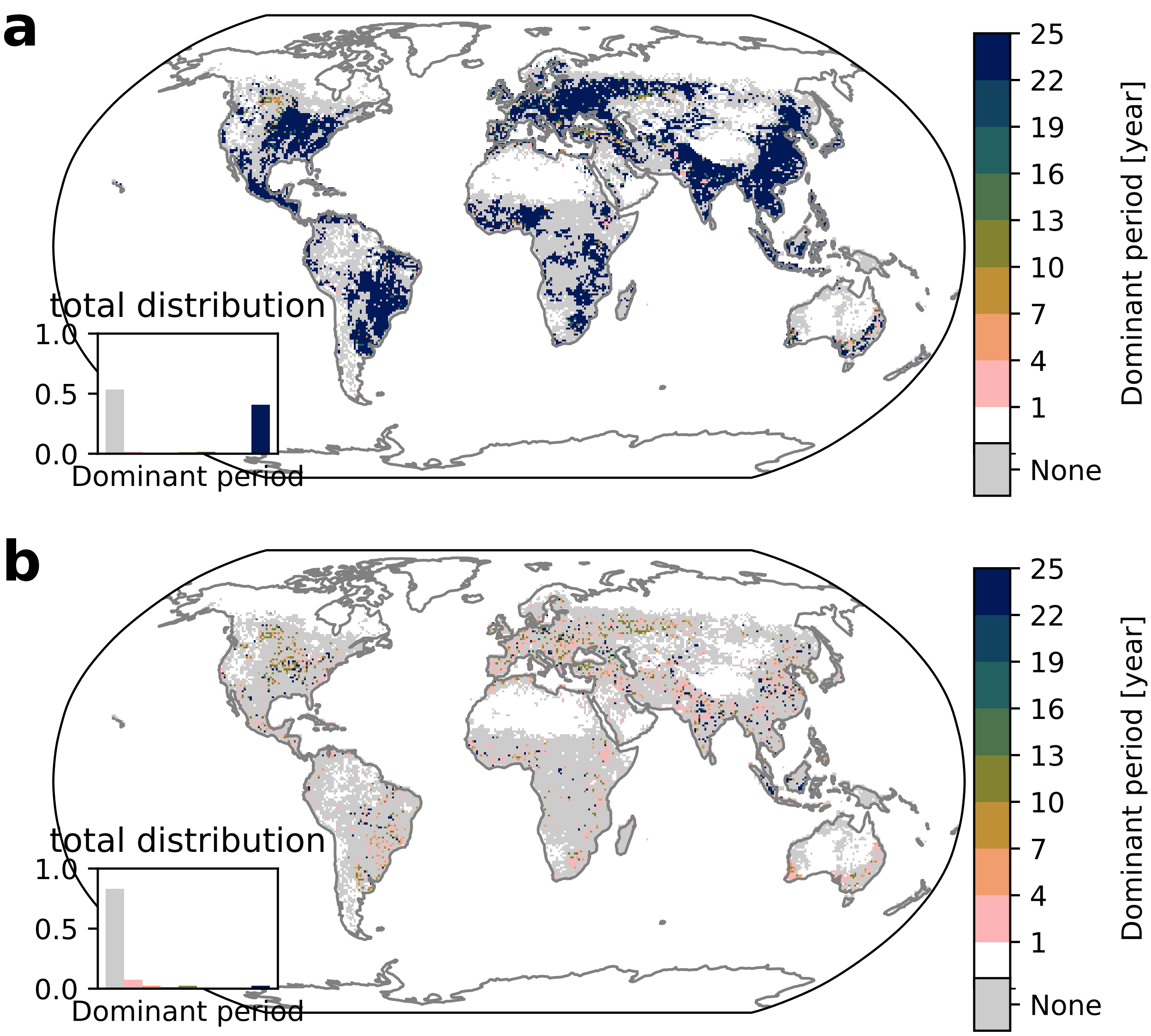

Supplementary Figure 29: **Dominant period for crop failure in 2050-2099.** Median dominant period for crop failure and (**a**) 2050-2099, and (**b**) linearly detrended 2050-2099 under SSP5-8.5. The white color signifies no extreme climate impact occurrence and gray color signifies no dominant period (irregularity) while existing dominant periods are grouped in three-year intervals ranging from 1-4 years to 22-25 years (blue). The inset shows the distribution of the dominant period counts.

In contrast, for heatwaves we find a similar distribution of dominant periods for heatwaves (see Supplementary Fig. 30 a) compared to the results in the main text (see Fig. 4 b in the main text). Intermediate dominant periods of 13-16 years typically occur at the boundary of highest and lowest dominant periods signifying an averaging effect between models still observing the strong warming trend while others already see yearly extreme heat events. Only in the case of detrended results we are not able to fully absorb the nonlinear trend over 2050-2099 which leads to vanishing dominant

periods in the detrended results (see Supplementary Fig. 30 b) while for the short time range 2040-2069 it is possible to sufficiently absorb the non-linear trend and recover highest dominant periods (see Fig. 4 c in the main text).

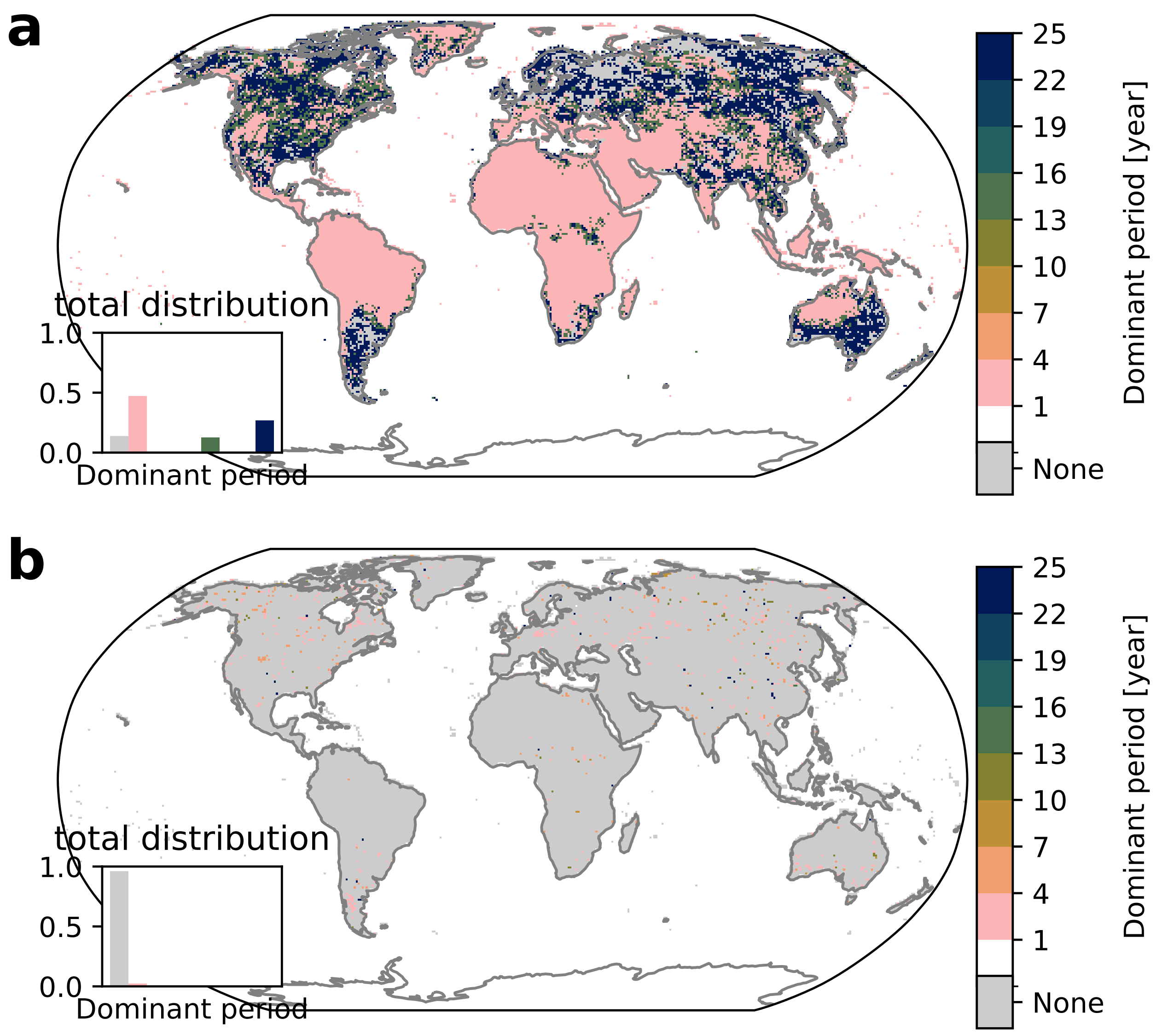

Supplementary Figure 30: **Dominant period for heatwave in 2050-2099.** Median dominant period for heatwaves and (**a**) 2050-2099, and (**b**) linearly detrended 2050-2099 under SSP5-8.5. The white color signifies no extreme climate impact occurrence and gray color signifies no dominant period (irregularity) while existing dominant periods are grouped in three-year intervals ranging from 1-4 years to 22-25 years (blue). The inset shows the distribution of the dominant period counts.

In the case of wildfires we find again the same effects as for crop failure and heatwaves with more irregularity than in 2040-2069 (compare Fig. 5 b in the main text) and a minor shift towards of smallest dominant periods through detrending (see Supplementary Fig. 31).

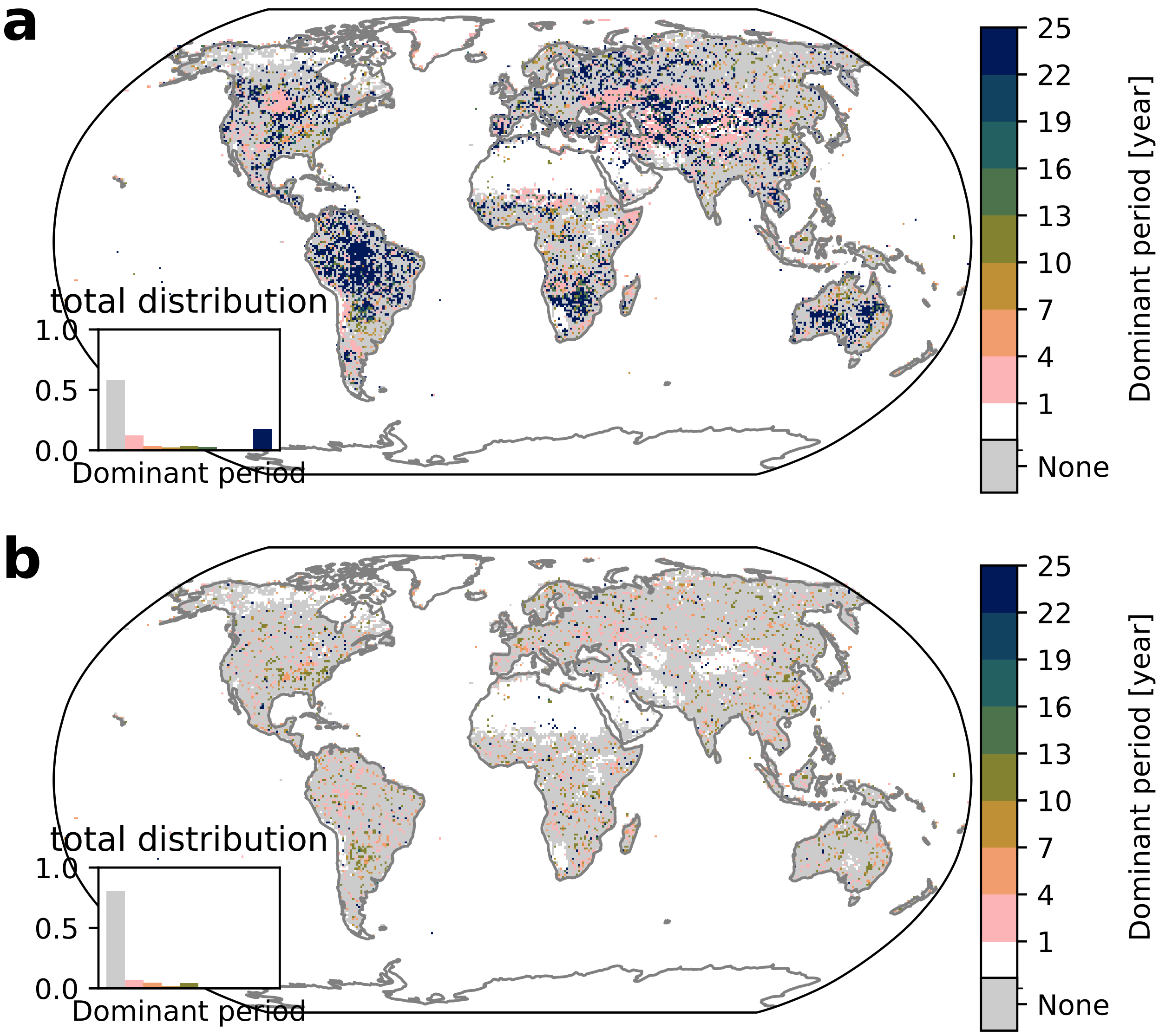

Supplementary Figure 31: **Dominant period for wildfire in 2050-2099.** Median dominant period for wildfire and (**a**) 2050-2099, and (**b**) linearly detrended 2050-2099 under SSP5-8.5. The white color signifies no extreme climate impact occurrence and gray color signifies no dominant period (irregularity) while existing dominant periods are grouped in three-year intervals ranging from 1-4 years to 22-25 years (blue). The inset shows the distribution of the dominant period counts.

10 Supplementary Discussion: Analysis with $2\Delta T = 250$ years

We investigate the effect of choosing the maximal time window, namely $2\Delta T = 250$ years, to account for large dominant periods that may be present in the extreme event data. The resulting median dominant periods for crop failure, heatwave, and wildfire affected areas for picontrol are shown in Supplementary Fig. 32.

In all three event categories there are almost no dominant return periods in contrast to the corresponding result with $2\Delta T = 50$ years (see Fig. 1 a in the main text). We attribute this irregularity to the large time window where temporal decorrelation occurs on the long time scale.

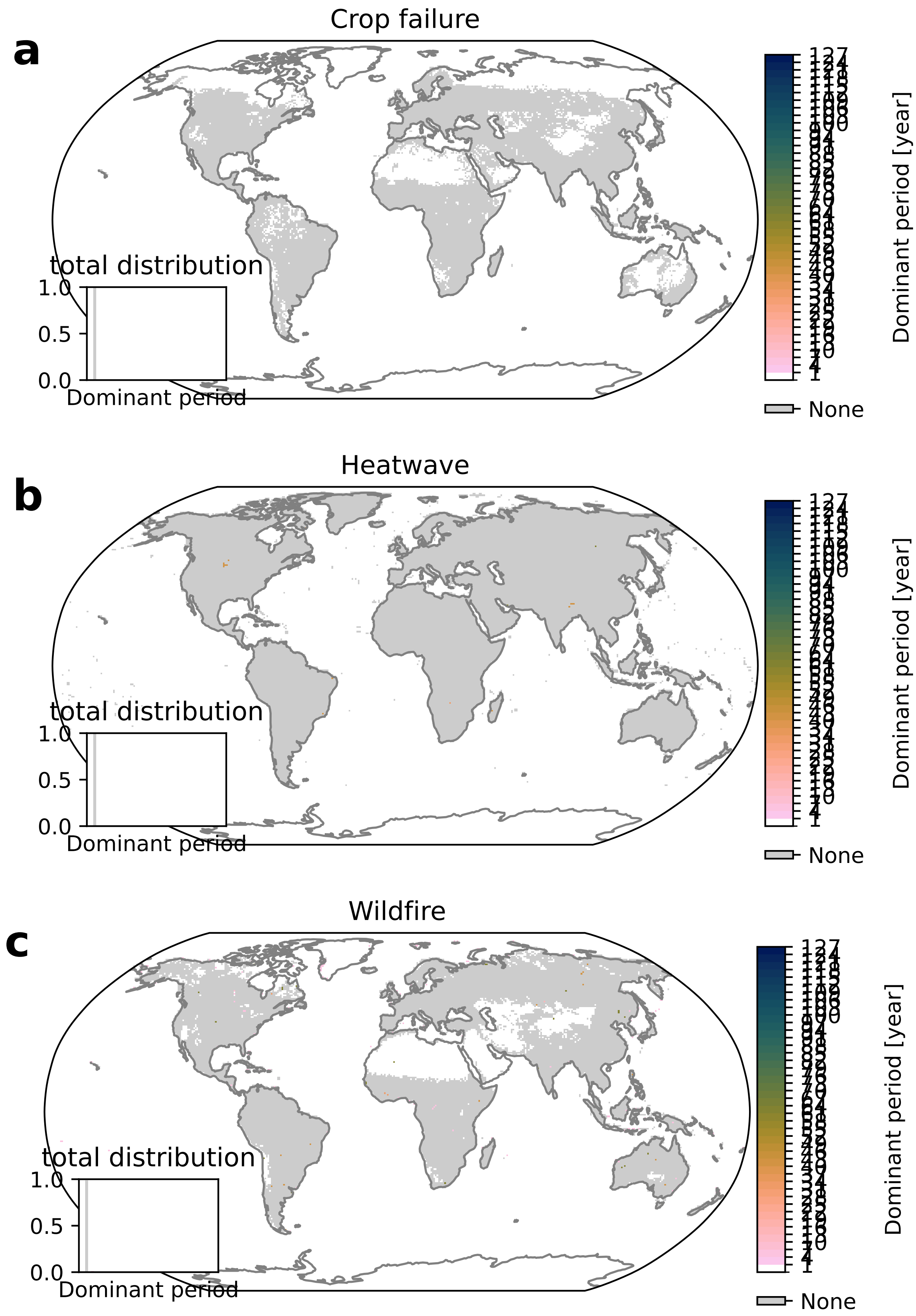

Supplementary Figure 32: **Dominant periods for** $2\Delta T = 250$**y.** Median dominant period in (**a**) crop failure, (**b**) heatwave, and (**c**) wildfire affected area at $t_0 = 1850$ for $2\Delta T = 250$ years under pre-industrial climate.

11 Supplementary Discussion: Comparison to autoregressive model

Here, we show the difference between the dominant period as calculated from Fourier analysis described in Sec. 4 with results from autoregressive models (AR). Since autoregressive models produce a continuous power spectrum we will compare to the strongest peak which corresponds to the strongest period. In addition, we estimate the optimal set of lag parameters for the autoregressive model through the AIC information criterion by comparing all possible model setups as implemented in ar_select_order method of the AR class in statsmodels [14]. We restrict the maximum lag of possible AR(n) models to 11 due to the exponential growth of possible model combinations.

	Dominant period (Fourier analysis)	Strongest period (Optimal AR(n) model)	Lags of the optimal AR(n) model
Example 1 (Fig. 7 a)	8.33y	15.53y	1, 2, 4, 5, 6, 7, 9, 10, 11
Example 2 (Fig. 7 b)	None (12.5y candidate)	6.88y	2, 3, 4, 6, 9, 10, 11
Example 3 (Fig. 7 c)	25y	7.63y	1, 3, 4, 5, 6, 8, 10

Supplementary Table 2: Comparison between dominant period and strongest period.

In the first example our method (see Sec. 4 step 4 in the main text) recognizes the smaller peak at time lag 8 and the relationship to the largest peak at time lag 16 as a higher harmonic period (see Supplementary Table 2). Consequently, the dominant period is given by 8 years, while the AR(n) model correctly identifies the largest peak at time lag 16 but there is no trivial way to connect this peak to the other spectral peaks (e.g. harmonics) of the continuous spectrum. The second example shows that both methods find different dominant periods in an ambiguous case, where peaks appear at 0, 13,14, and 21 years lag. These peaks are compatible with dominant periods of 12.5 and 6.88 years where in both cases one peak is misplaced (21 instead of 25) or missing (6.88 missing). In the case of 12.5 years the significance test for red noise fails in this ambiguous case which is why our method finds no dominant period. In the last example we find different periods due to differences in the spectral estimates. The largest spectral density in Fourier analysis is found to be at 25y period in contrast to 7.63 years from the AR model. This difference stems from the trend in the time series which is partly absorbed across coefficients of the AR model while the Fourier analysis absorbs the trend mostly in the largest dominant period.

12 Supplementary Discussion: Standard deviation of dominant periods

We present the deviation between dominant periods from different model combinations and time windows in terms of the pinkviation within the picontrol setup. Note that the standard deviation is calculated between all model combinations that detect a dominant period. We find small standard deviations mostly concentrated between 0 and 4 across all event categories (see Supplementary Fig. 33). While we find little deviation between the climate model and time window results we still do not observe a median dominant period due to the minimal time window restriction (see Sec. 4.3 in the main text).

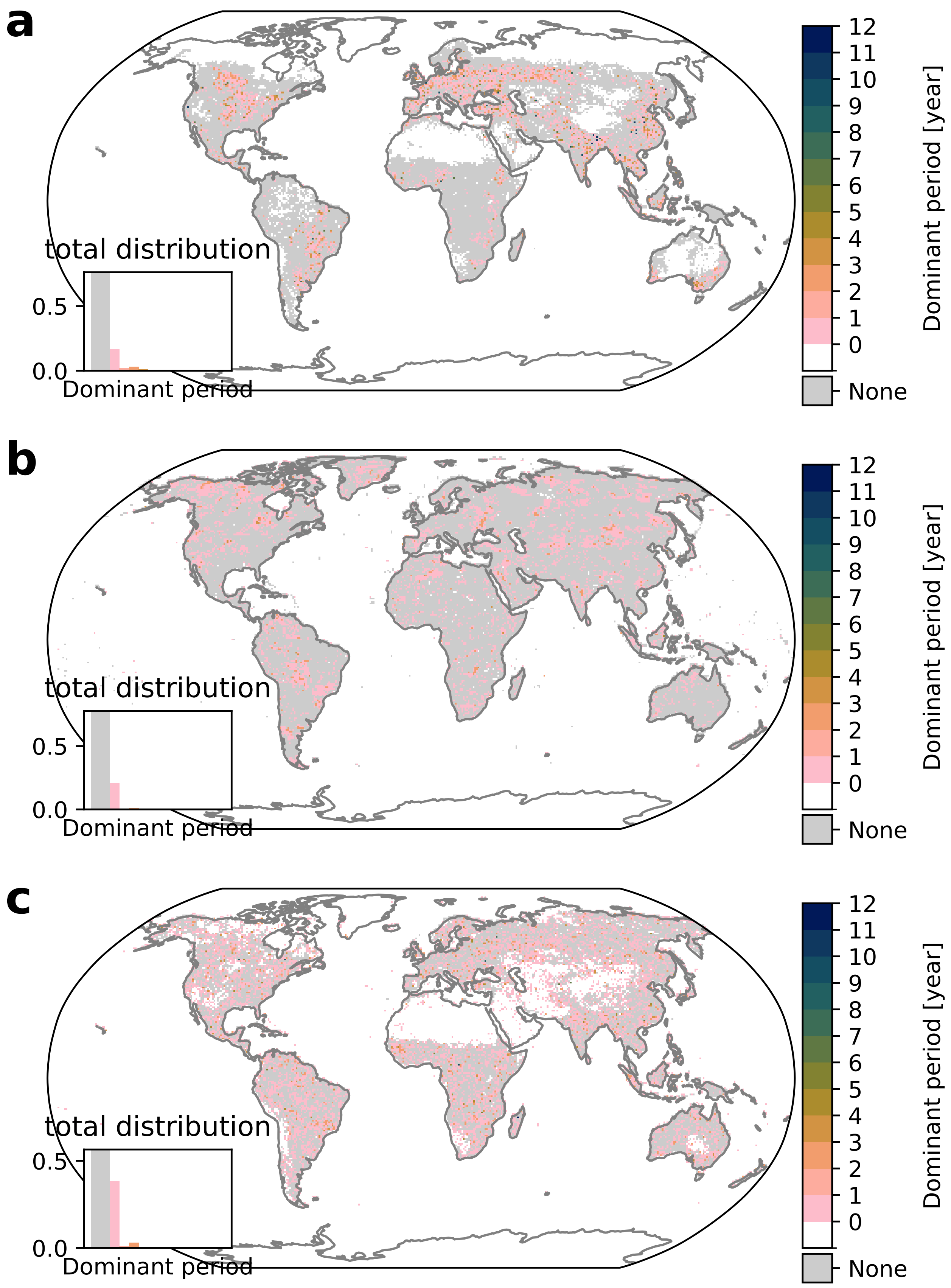

Supplementary Figure 33: **Standard deviation of dominant periods.** Standard deviation of dominant periods for (**a**) crop failure, (**b**) heatwave, and (**c**) wildfire for picontrol aggregated over all time windows 1850-1899, 1900-1949, . . ., 2050-2099 and climate-impact models. The white color signifies no extreme climate impact occurrence and gray color signifies no dominant period (irregularity) while existing dominant period standard deviations range from 1 year (pink) to 12 years (blue). The inset shows the distribution of the dominant period standard deviation counts.

Supplementary References

1. Grandey, B. S. *et al.* Monte Carlo drift correction – quantifying the drift uncertainty of global climate models. *Geoscientific Model Development* **16,** 6593–6608 (2023).
2. Song, G. & Ren, R. The Subsurface and Surface Indian Ocean Dipoles and Their Association with ENSO in CMIP6 models. *Advances in Atmospheric Sciences* **40,** 975–987 (2023).
3. Frieler, K. *et al.* Scenario setup and forcing data for impact model evaluation and impact attribution within the third round of the Inter-Sectoral Impact Model Intercomparison Project (ISIMIP3a). *Geoscientific Model Development* **17,** 1–51 (2024).
4. Heino, M. *et al.* Two-thirds of global cropland area impacted by climate oscillations. *Nature Communications* **9,** 1257 (2018).
5. Anderson, W. B., Seager, R., Baethgen, W., Cane, M. & You, L. Synchronous crop failures and climate-forced production variability. *Science Advances* **5,** eaaw1976 (2019).
6. Heino, M., Guillaume, J. H. A., Müller, C., Iizumi, T. & Kummu, M. A multi-model analysis of teleconnected crop yield variability in a range of cropping systems. *Earth System Dynamics* **11,** 113–128 (2020).
7. Trenberth, K. *Climate Analysis Section, NCAR, Boulder, USA, Trenberth (1984). Updated regularly. Accessed 01 August 2025*
8. Iizumi, T. *et al.* Impacts of El Niño Southern Oscillation on the global yields of major crops. *Nature Communications* **5,** 3712 (2014).
9. Laris, P. & Dembele, F. Humanizing savanna models: integrating natural factors and anthropogenic disturbance regimes to determine tree–grass dynamics in savannas. *Journal of Land Use Science* **7,** 459–482 (2012).
10. Archibald, S., Lehmann, C. E. R., Gómez-Dans, J. L. & Bradstock, R. A. Defining pyromes and global syndromes of fire regimes. *Proceedings of the National Academy of Sciences* **110,** 6442–6447 (2013).
11. Krawchuk, M. A., Moritz, M. A., Parisien, M.-A., Van Dorn, J. & Hayhoe, K. Global Pyrogeography: the Current and Future Distribution of Wildfire. *PLOS ONE* **4,** 1–12 (Apr. 2009).
12. Artés, T. *et al.* A global wildfire dataset for the analysis of fire regimes and fire behaviour. *Scientific Data* **6,** 296 (2019).
13. Chen, Y. *et al.* A pan-tropical cascade of fire driven by El Niño/Southern Oscillation. *Nature Climate Change* **7,** 906–911 (2017).
14. Seabold, S. & Perktold, J. *statsmodels: Econometric and statistical modeling with python* in *9th Python in Science Conference* (2010).
